# Photochemical Ring-Opening Reaction of 1,3-Cyclohexadiene: Identifying the True Reactive State

**DOI:** 10.1021/jacs.2c06296

**Published:** 2022-11-29

**Authors:** Oksana Travnikova, Tomislav Piteša, Aurora Ponzi, Marin Sapunar, Richard James Squibb, Robert Richter, Paola Finetti, Michele Di Fraia, Alberto De Fanis, Nicola Mahne, Michele Manfredda, Vitali Zhaunerchyk, Tatiana Marchenko, Renaud Guillemin, Loic Journel, Kevin Charles Prince, Carlo Callegari, Marc Simon, Raimund Feifel, Piero Decleva, Nad̵a Došlić, Maria Novella Piancastelli

**Affiliations:** † Sorbonne Université, CNRS, Laboratoire de Chimie Physique-Matière et Rayonnement, LCPMR, ParisF-75005, France; ‡ Institut Rud̵er Bošković, ZagrebHR-10000, Croatia; § Department of Physics, University of Gothenburg, GothenburgSE-412 96, Sweden; ∥ Elettra-Sincrotrone Trieste, Trieste34149, Italy; ⊥ European XFEL, SchenefeldD-22869, Germany; # IOM-CNR, S.S. 14 km 163.5 in Area Science Park, Trieste34149, Italy; ¶ Dipartimento di Scienze Chimiche e Farmaceutiche, Universitá di Trieste, TriesteI-34127, Italy; ∇ Department of Physics and Astronomy, Uppsala University, UppsalaSE-751 20, Sweden

## Abstract

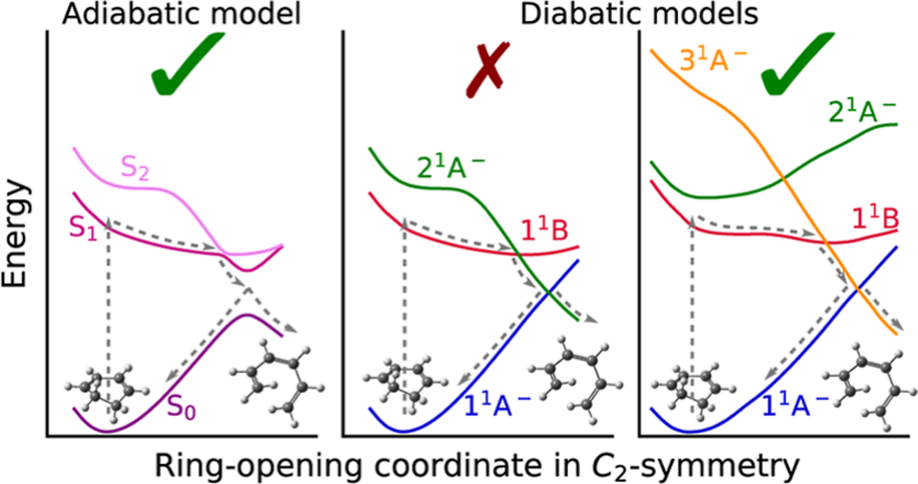

The photochemically induced ring-opening isomerization reaction of 1,3-cyclohexadiene to 1,3,5-hexatriene is a textbook example of a pericyclic reaction and has been amply investigated with advanced spectroscopic techniques. The main open question has been the identification of the single reactive state which drives the process. The generally accepted description of the isomerization pathway starts with a valence excitation to the lowest lying bright state, followed by a passage through a conical intersection to the lowest lying doubly excited state, and finally a branching between either the return to the ground state of the cyclic molecule or the actual ring-opening reaction leading to the open-chain isomer. Here, in a joint experimental and computational effort, we demonstrate that the evolution of the excitation–deexcitation process is much more complex than that usually described. In particular, we show that an initially high-lying electronic state smoothly decreasing in energy along the reaction path plays a key role in the ring-opening reaction.

## Introduction

The photochemical ring-opening reaction of 1,3-cyclohexadiene (CHD) to 1,3,5-hexatriene (HT) is a textbook example of a pericyclic reaction.^1,2^ In addition to many theoretical investigations,^3−13^ the dynamical evolution of photoexcited CHD has been studied, possibly more than any other photochemical reaction, with a large variety of spectroscopic techniques for isolated molecules, that is in collision-free conditions and without solvent effects (see e.g., refs (14)–^22^). This pronounced interest arises from the fundamental importance of the reaction, its biological relevance,^23,24^ and a range of applications in organic synthesis and materials science.^25−29^

The conceptual framework to understand the photochemistry of CHD is provided by the Woodward–Hoffmann rules,^30^ extended by van der Lugt and Oosterhoff,^31,32^ stating that the conrotatory ring-opening reaction is mediated by a doubly excited electronic state of the same symmetry as the ground state (1^1^A^–^). In the ring-opening reaction, the lowest unoccupied molecular orbital of CHD, which is doubly occupied in the reactive state, becomes the highest occupied molecular orbital of HT. The generally accepted sequence of events starts with a valence excitation by a wavelength of about 267 nm to the first (S_1_) bright state, labeled 1^1^B, followed by a passage through a conical intersection (CoIn) to a dark state, labeled 2^1^A^–^. The following step is a branching between two pathways at a second CoIn, either the return to the ground state of the cyclic molecule or the actual ring-opening reaction leading to the open-chain isomer (see e.g. ref (11)–^15^). It is noticed that the labels above and throughout the text indicate diabatic states and that we use the notation for alternant π-system (plus and minus) pseudosymmetry.^33,34^

In general, photochemical reactions are assumed to be driven by CoIns at which two adiabatic Born–Oppenheimer potential energy surfaces (PESs) become degenerate and which act as effective funnels for the transfer of population between different adiabatic PESs.^35−37^ In contrast to adiabatic PESs, which suddenly change their chemical character near a CoIn, PESs that retain their character and cross at CoIns are known as diabatic states. As properties such as the electronic transition dipole moment change smoothly only in the diabatic representation, the dynamics of the diabatic electronic population is the one monitored in time-resolved ultrafast spectroscopy.^38−41^

Despite tremendous efforts, a conclusive proof of the reactivity of the diabatic 2^1^A^–^ state has not yet been achieved. To address this problem, we ask a seemingly simple question: is there any other electronic state of symmetry A and partial double excitation character that may be involved in the ring-opening reaction in CHD? Here, in a joint experimental and computational effort, we show that the evolution of the process is more complex than that usually described. In particular, we show that a high-lying state with a pronounced double excitation character, labeled 3^1^A^–^, plays a key role in the ring-opening reaction.

To observe the first stages of a photochemical process, a suitable method is to prepare a photoexcited state with an optical laser, the so-called pump, and then follow its evolution by valence photoelectron spectroscopy, the so-called probe, as a function of pump–probe time delay, with resolution on the picosecond (ps, 10^–12^ s) or femtosecond (fs, 10^–15^ s) timescale. Time-resolved photoemission is the first-choice technique to follow the evolution of a system as it provides information on both electronic and nuclear dynamics. Furthermore, it provides information on states which are not reachable by absorption methods, in particular dark states, which need to be characterized in the present case.

A breakthrough in this direction is represented by the FERMI free-electron laser (FEL) at the Elettra facility, Trieste, Italy. Time-resolved photoemission spectra can be obtained here with the spectral resolution high enough to precisely characterize ionization from electronic states even if they are weak and/or close in energy (see e.g., refs (42) and (43)). FERMI, as a seeded FEL, offers the advantages of a narrow photon energy bandwidth, negligible photon energy jitter, higher stability, higher pulse energies, and much higher photon fluxes with respect to monochromatized sources based on high harmonic generation.

## Results and Discussion

Our experiments were performed at FERMI at the Low Density Matter (LDM) beamline, devoted to atomic and molecular spectroscopy studies.^44^ The pump was a titanium–sapphire optical laser, providing a wavelength of 267 nm, and the probe was valence photoelectron spectroscopy with a photon energy of 19.23 eV. The delay time range was from −1 to 2 ps, spanned in steps of 50–100 fs. We recorded the valence photoelectron spectra with a magnetic bottle spectrometer^45,46^ (see Supporting Information, SI, for further details on the facility, the beamline, and the spectrometer).

**Figure 1:**
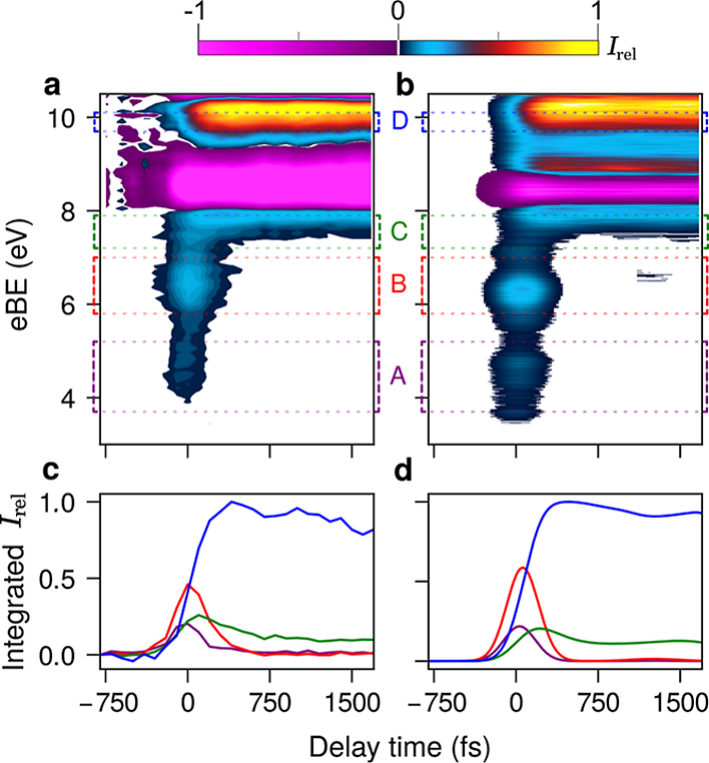
(a) Experimental and (b) computed differential 2D maps of the photoelectron spectra of CHD. White color denotes the regions with . For the computed excited-state absorption component of the signal (separated on CHD- and HT-ending trajectories), check Figure S5. (c) Experimental and (d) computed time evolution of the spectral intensity integrated over the area marked by colored rectangles in (a). For experimental and computational details, see the Supporting Information.

In the upper panels (experiment and theory) of Figure 1, we show the valence photoelectron spectra recorded for several values of pump–probe delay in the delay range from −1 to 2 ps in steps of 100 fs. The spectra are plotted as a two-dimensional map, highlighting the variations in spectral intensity as a function of time delay. The ground-state spectrum is subtracted from the spectra obtained at later times (see Supporting Information and Figures S2–S4 for further details on how the experimental spectra are obtained). The upper panels illustrate the variations of the photoionization signal as a function of the pump–probe delay, and the changes of averaged signals in the four characteristic areas are shown in the lower panels (experiment and theory). We notice the development of a series of new features in two electron energy regions, that is, at a low binding energy (4–8 eV) and around 10 eV. The low-binding-energy region is characteristic of spectral features related to excited states or dynamical features due to the photoexcitation process, while the 10 eV region is in the range characteristic of ground-state features.

The three features in the low-binding-energy region have a different behavior as a function of delay: the two peaks at 4.5 and 6.4 eV binding energies reach maxima at 0 ps and decrease to zero intensity after 0.5 ps, while the third feature at 7.5 eV has a low intensity at 0 ps, continues to grow to a maximum at 0.5 ps, and finally stabilizes after about 1 ps. The feature at a high binding energy, 9.9 eV, grows after 0 ps as a function of delay and stays constant after about 0.5 ps. We denote these features A, B, C, and D in the order of increasing binding energy. The disappearing features A and B “feed” the developing ones C and D, giving evidence of a dynamical change accompanying the electronic relaxation. This is a first hint that features A and B correspond to excited states which relax on a femtosecond timescale, while the features C and D that develop and then reach an asymptotic value are connected to the electronic states belonging to vibrationally hot open chain and/or closed ring isomers.

This assignment is strongly supported by theory. The photoinduced dynamics was simulated with nonadiabatic surface-hopping trajectories. In this method, an ensemble of classical trajectories is propagated starting from the excited state, and the stochastic fewest switches algorithm^47^ is used to allow each trajectory to “hop” to different electronic states based on the nonadiabatic couplings. A total of 107 trajectories were propagated for 1000 fs, with a time step of 0.5 fs in a manifold of three electronic states. The energies and forces needed for the propagation of the trajectories are computed using the (XMS(3)-CASPT2(6,6)) method, whose accuracy was assessed in ref (12) (see the Computational Section and Supporting Information for details).

The photoelectron spectra were computed in the sudden approximation in which the partial cross sections are approximated with Dyson orbital norms.^48−50^ The accuracy of the sudden approximation was assessed by comparison with the benchmark spectrum computed using a B-spline description of the photoelectron continuum^51,52^ (see Figure S6). To match the experiment, the theoretical spectra have been shifted by +0.3 eV. For further details on how the theoretical spectra are calculated, see the Methods section and Supporting Information. The agreement between the experimental and simulated spectra is remarkably good, both in terms of intensity and time evolution of the peaks, with the width of the ground-state bleach component (purple) being the only major discrepancy. The simulated spectra, when analyzed separately for nonreactive (CHD) and reactive (HT) trajectories (see Figure S5), show that band C arises mainly from the vibrationally hot CHD, while band D is primarily due to the newly formed HT molecules. The latter assignment is confirmed by a comparison between the ground-state valence photoelectron spectra of CHD and HT, where there is spectral intensity in the binding energy region around 10 eV only for the open-chain isomer.^53,54^

While the assignment of features C and D is rather straightforward, the behavior of features A and B, which correspond to the excited states and therefore are the key to identify the true reactive one, is more complex. A first hint of the three-state model reported in the literature being oversimplified is that we do not observe a simple series of few peaks growing and decreasing in temporal sequence, as, for example, was reported in ref (42) for acetylacetone, where the adiabatic description was sufficient.

To fully explain the mechanism of the reaction, we need to connect the evolution of the photoionization bands as a function of time with the population of the diabatic electronic states involved in the reaction. To this end, we first consider the properties of the electronic states at the Franck–Condon geometry. The electronic spectrum of CHD reported in the literature is composed of two broad bands at ∼5.0 and ∼8.0 eV.^55^ The first encompasses the bright 1^1^A^–^ → 1^1^B and dark 1^1^A^–^ → 2^1^A^–^ transitions. The second or *cis*-band is characteristic for *cis*-polyenes. Here, two valence transitions of mixed character have been identified—the intense 1^1^A^–^ → 1^1^A^+^ transition at 8.0 eV^4,56^ and a higher lying transition with a considerable vibronic structure.^55^ Intercalated between these two bands are several sharp Rydberg transitions.^4,57^ Owing to their weak coordinate dependence, Rydberg states are expected to play a negligible role in the ring-opening reaction.^58^

Vertical excitation energies of the six lowest valence excited states of CHD with the coefficients of the leading configuration state functions (CSFs) are collected in Table 1 (for EOM-CC3, results see Table S1), while the orbitals constituting the active space are illustrated in Figure S7.

**Table 1:** State Ordering and Vertical Excitation Energies (in eV) of the Six Lowest Valence-Excited States at the Franck–Condon Geometry Labeled According to Plus–Minus Alternancy Symmetry, Leading CSFs and Their CI Coefficients Being Calculated at the XMS(7)-CASPT2[6e,6o] Level of Theory^a^

state	*E*/eV (*f*)	exp.^b^*E*/eV	leading CSFs	CI coeff.
1^1^A^–^			Aufbau	0.96
			π_2_π_2_ → π_1_*π_1_*	–0.14
1^1^B	5.18 (0.017)	4.94	π_2_ → π_1_*	0.97
2^1^A^–^	5.99 (0.008)		π_2_π_2_ → π_1_*π_1_*	0.53
			π_1_ → π_1_*	–0.55
			π_2_ → π_2_*	0.46
2^1^B	7.50 (0.007)		σ → π_1_*	0.97
1^1^A^+^	8.31 (0.321)	7.90	π_1_ → π_1_*	0.69
			π_2_ → π_2_*	0.65
			π_2_π_2_ → π_1_*π_1_*	0.16
3^1^A^–^	8.66 (0.217)		π_2_π_2_ → π_1_*π_1_*	0.68
			π_2_ → π_2_*	–0.51
			π_1_ → π_1_*	0.28
3^1^B	10.11 (0.000)		π_1_ → π_2_*	0.96

a For EOM-CC3 states, see Table S1.

b Optical and electron energy loss spectroscopies.^56^

**Figure 2:**
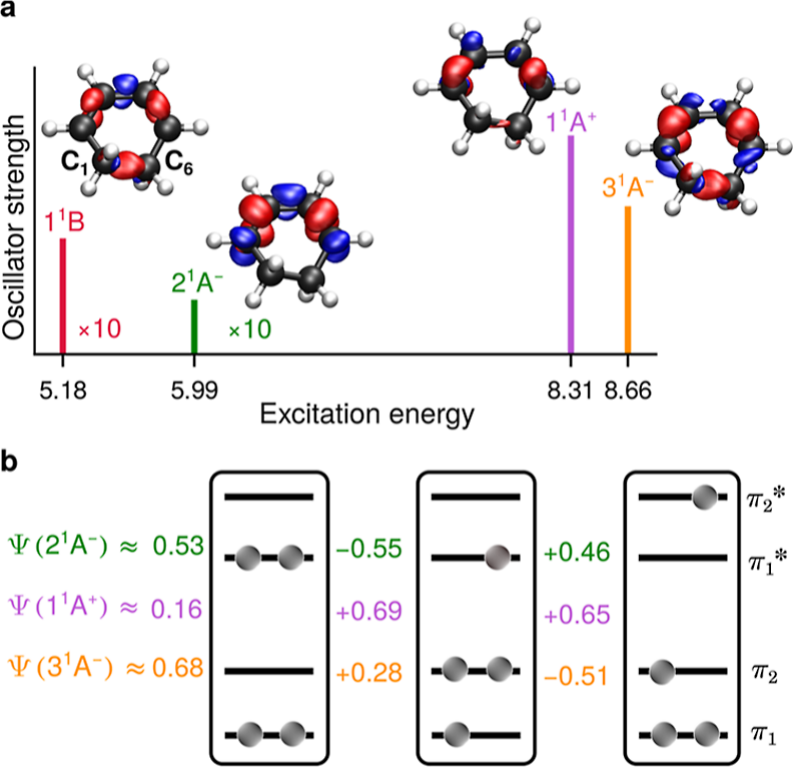
(a) Excitation energies and relative absorption intensities of the 1^1^B, 2^1^A^–^, 1^1^A^+^, and 3^1^A^–^ states (sticks) and the corresponding maps showing the electron density difference with respect to the electronic ground state calculated at the Franck–Condon geometry. Areas of increased and reduced electron densities are shown in blue and red, respectively. (b) Three valence states, 2^1^A^–^ (green), 1^1^A^+^ (purple), and 3^1^A^–^ (orange), with the CI coefficients of the three dominant CSFs (π_2_π_2_ → π_1_^*^π_1_^*^, π_1_ → π_1_^*^, and π_2_ → π_2_^*^).

The main results are summarized in Figure 2. At the Franck–Condon geometry, the XMS(7)-CASPT2[6e, 6o] method yields the 1^1^B state at 5.18 eV, the dark 2^1^A^–^ state at 5.99 eV, and the two bright states of the *cis-*band, 1^1^A^+^ and 3^1^A^–^, at 8.31 and 8.66 eV, respectively. The electron density differences between the excited states and the ground state plotted in Figure 2a show that excitation to the 1^1^B and 3^1^A^–^ states leads to the depletion of electron density on the C_1_–C_6_ bond, while excitation to 2^1^A^–^ and 1^1^A^+^ leaves the density on that bond nearly unchanged. From the wave functions of the three states of symmetry A, which are dominated by the three CSFs shown in Figure 2b, it is evident that the 3^1^A^–^ state has a pronounced double excitation character (see CI coefficients) and the overall electronic properties of a potentially reactive state.

**Figure 3:**
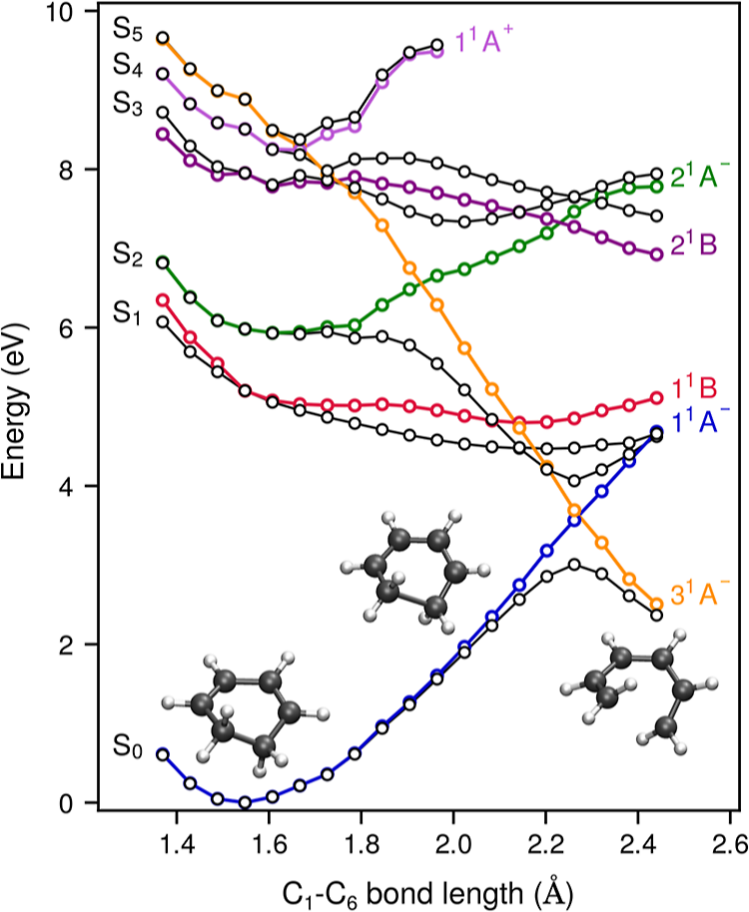
Coordinate dependence of the potential energy of the lowest adiabatic electronic states (black) and diabatic electronic states: 1^1^A^–^ (blue), 1^1^B (red), 2^1^A^–^ (green), 2^1^B (dark violet), 1^1^A^+^ (light violet), and 3^1^A^–^ (orange). For comparison with the traditional three-state model, see Figure S8.

Figure 3 shows the one-dimensional potential energy profiles of the adiabatic (black) and diabatic (colors) states along the ring-opening path in C_2_ symmetry. The diabatization has been performed on the basis of the lowest six adiabatic states by minimizing the variation of their wave functions along the reaction path.^59^ The diabatization on the usual basis of the three lowest diabatic states, as defined at the FC geometry, is shown in Figure S8 (see also Figure S9). Details of the procedure are given in the Supporting Information. One sees that the coordinate dependence of the diabatic 1^1^B state (red) matches the expected behavior, but this is not the case for the 2^1^A^–^ state (green), which clearly increases in energy (destabilizes) along the reaction path. The diabatic 3^1^A^–^ state (orange) is the state that is strongly stabilized in the reaction. The two states cross at *R*(C_1_–C_6_) ∼ 1.9 Å where the adiabatic S_2_ state shows the characteristic inflection. Further down the reaction path, 3^1^A^–^ crosses with the 1^1^B state and at *R*(C_1_–C_6_) ∼ 2.3 Å with the ground state. This indicates that the ground state of HT correlates with the 3^1^A^–^ state and not with the 2^1^A^–^ state. Notice that the proposed pathway does not contradict the Woodward–Hoffmann rules as they do not prescribe that the reaction should proceed on the lowest doubly excited state at the FC geometry.

**Figure 4:**
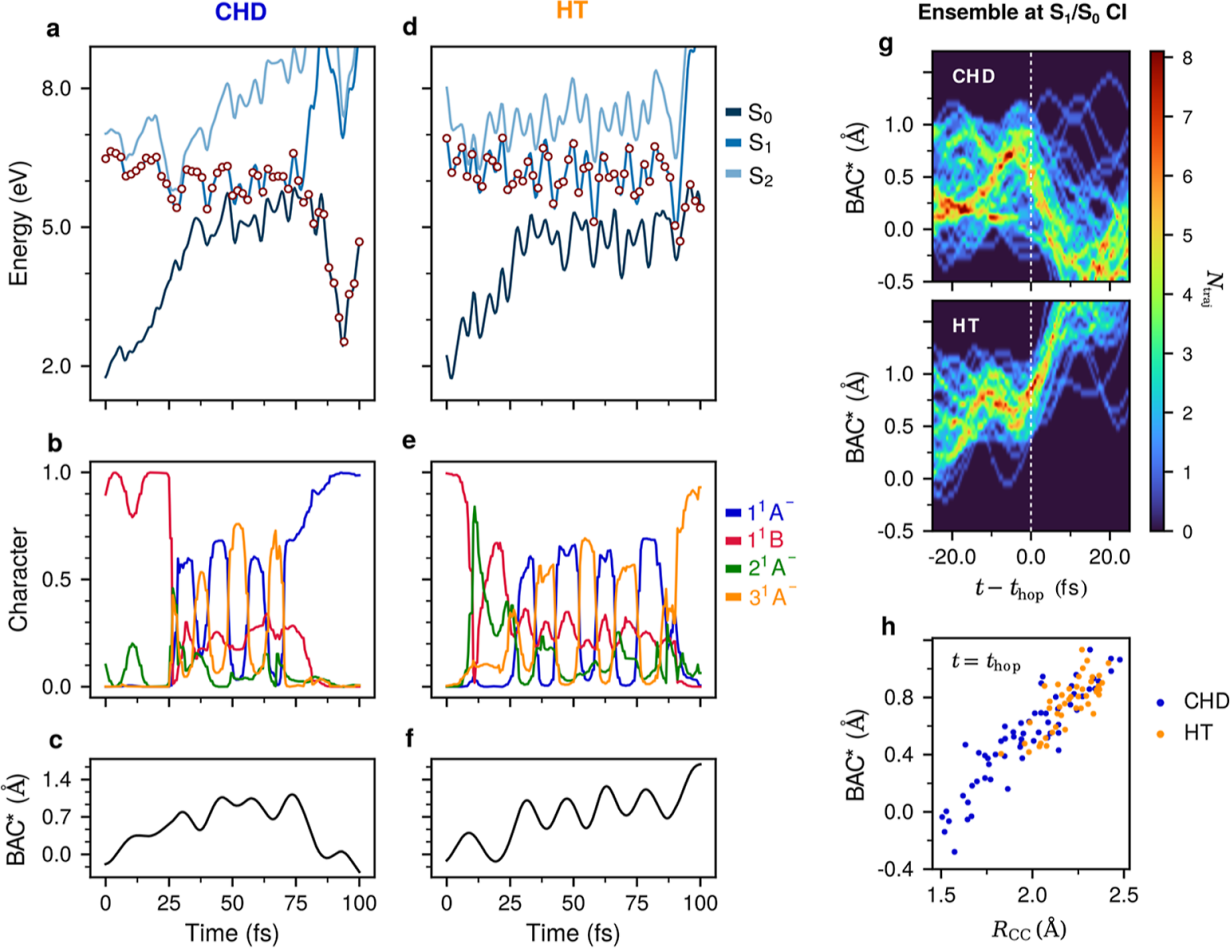
Two representative nonadiabatic trajectories leading to CHD (a–c) and HT (d–f). (a,d) Time evolution of the potential energy of the electronic ground state S_0_ (dark blue) and the two lowest excited states S_1_ (blue) and S_2_ (light blue). Dots mark the instantaneous populations of electronic states. (b,e) Decomposition of the currently populated state in terms of four diabatic states, 1^1^A^–^ (blue), 1^1^B (red), 2^1^A^–^ (green), and 3^1^A^–^ (orange), along the trajectories. (c,f) Time evolution of the BAC* along the nonadiabatic trajectories. (g) Evolution of the BAC* coordinate for the ensemble of nonadiabatic trajectories synchronized to reach the S_1_/S_0_ CoIn simultaneously at *t*′ = *t* – *t*_hop_ = 0. At the moment of hop, BAC* decreases for CHD and increases for HT trajectories. For the evolution of the *R*(C_1_–C_6_) coordinate and relative velocity, d*R*(C_1_–C_6_)/d*t*, see Figure S13. (h) Distribution of *R*(C_1_–C_6_) and BAC* at the moment of hop to S_0_. Orange (blue) circles correspond to HT (CHD) trajectories. For other structural parameters, see Figure S14.

To understand the role of the different diabatic states in the ring-opening reaction when the C_2_ symmetry is lifted, we analyze two representative nonadiabatic trajectories yielding CHD (Figure 4a–c) and HT (Figures 4d–4f). For additional trajectories, see Figure S10. The two trajectories exhibit a very similar behavior up to the CoIn with the ground state. In both cases, the dynamics is initiated in the S_1_ state (Figure 4a,d), which is in the diabatic 1^1^B state (Figure 4b,e). At two instances, at ∼10 and ∼25 fs, the gap between the S_1_ and S_2_ states becomes vanishingly small, and the S_1_ state has a possibility to change its character.

**Figure 5:**
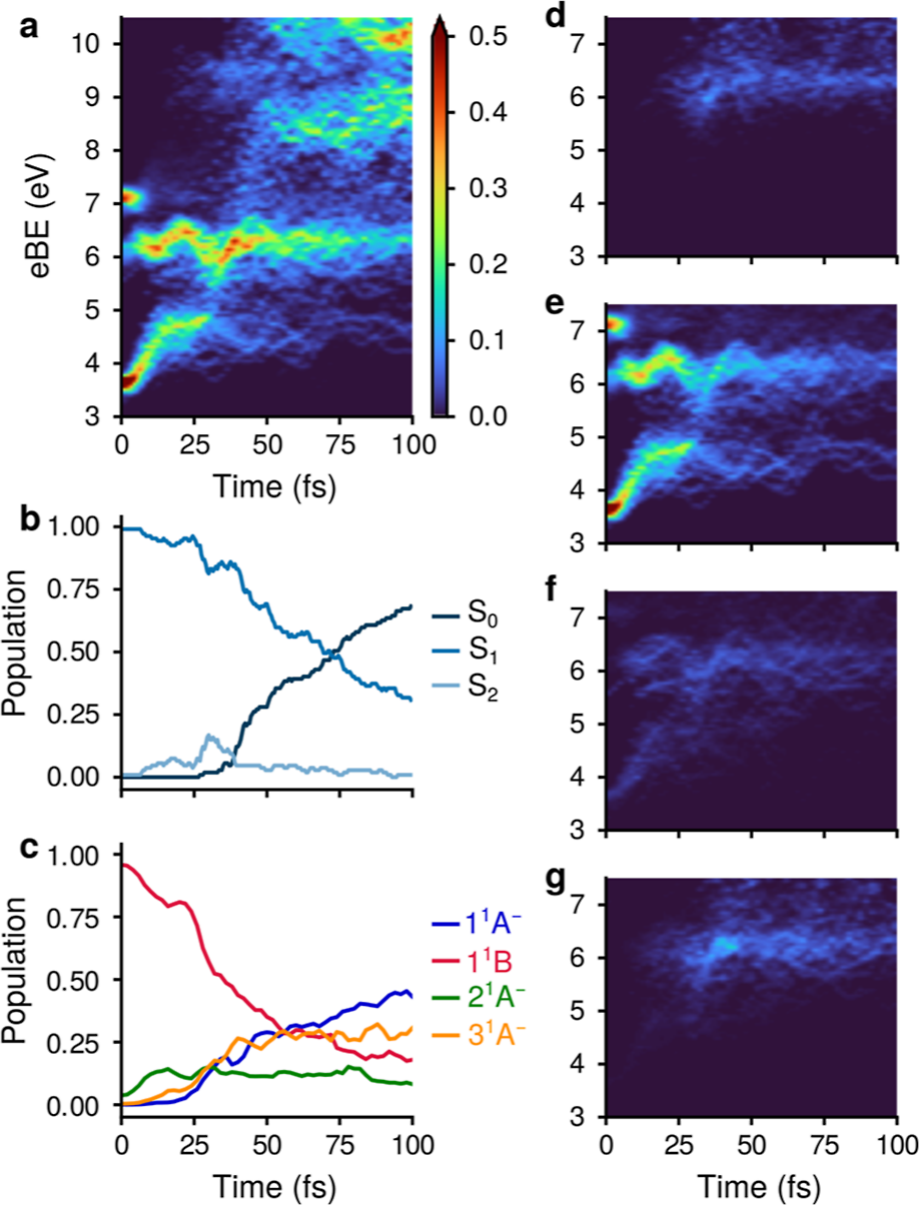
(a) Unconvoluted photoelectron spectra for short delay times. The ground-state bleach component is not taken into account. (b) Time evolution of the adiabatic population of electronic states obtained from surface-hopping nonadiabatic dynamics simulations. (c) Time evolution of the diabatic population of electronic states obtained by diabatization of electronic states along nonadiabatic trajectories. (d–g) Decomposition of the photoionization spectrum in terms of contributions of the diabatic states 1^1^A^–^ (d), 1^1^B (e), 2^1^A^–^ (f), and 3^1^A^–^ (g).

Indeed, the diabatic populations show a brief increase of the 2^1^A^–^ contribution at ∼10 fs (green), but then the 1^1^B character is recovered until ∼25 fs when its contribution suddenly drops. From ∼25 fs onward, the S_1_ state is best described as a superposition of the 1^1^A^–^ (blue) and 3^1^A^–^ (orange) diabatic states. The two states are strongly coupled by nuclear motion along the so-called extended bond-alternating coordinate (BAC*), which is the difference of single and double bond lengths in HT plus *R*(C_1_–C_6_).^11^ In the CHD trajectory, the return to the ground state occurs after a BAC* local maximum, while the C_1_–C_6_ bond is compressing and the S_1_ state is dominantly of 1^1^A^–^ character. On the contrary, in the HT trajectory, the CoIn with the ground state is encountered after a BAC* local minimum, while the C_1_–C_6_ bond is expanding and the S_1_ state is dominantly of 3^1^A^–^ character. After the hop, the CHT trajectory continues to evolve in the 1^1^A^–^ state, and the HT trajectory continues to evolve in the 3^1^A^–^ state, which is now the ground state leading to the HT product. Altogether, our analysis suggests that the fate of a nonadiabatic trajectory is determined by the character of the S_1_ state at the moment of the S_1_→S_0_ nonadiabatic transition. If the transition occurs when the S_1_ state has a dominant 1^1^A^–^ character, the CHD product is formed and, vice versa, if the hop occurs when the S_1_ state has a dominant 3^1^A^–^ character, the HT product is formed. The analysis of the ensemble of nonadiabatic trajectories, divided in two groups, CHD and HT, and synchronized in such a way as to reach the S_1_/S_0_ CoIn at the same time, is given in Figure 4g,h. Figure 4g shows that for all HT trajectories BAC* increases before the hop to S_0_, while it decreases for most but not all CHD trajectories. The distribution of *R*(C_1_–C_6_) and BAC* at the time of hop (Figure 4h) indicates that for large BAC* and *R*(C_1_–C_6_) distances, both CHD (blue) and HT (orange) can be formed in a close to 50:50 ratio, but for small BAC* and short *R*(C_1_–C_6_), only CHD is formed, irrespective of whether BAC* is compressing or not. A closer inspection reveals that in this group of nonreactive trajectories, the population of the 3^1^A^–^ state is negligibly small (see Figure S11), meaning that the existence of a second nonreactive pathway from either 1^1^B or 2^1^A^–^ cannot be excluded. We can now relate the average adiabatic and diabatic electronic populations to the time evolution of the two lowest energy bands in the photoelectron spectra. Figure 5a shows the two-dimensional map of theoretical photoelectron spectra at short delay times, as computed using eq 4 in the Supporting Information (without broadening the calculated signal to match the experimental resolution).

Two bands are clearly visible in the binding energy range of 3–7 eV. Band A starts at ∼3.2 eV, and within 15 fs reaches a plateau at ∼4.5 eV. It arises from the S_1_→D_0_ transition. The increase of the ionization energy is caused by the motion toward the minimum of the S_1_ state. As this motion leads to the extension of the C_1_–C_6_ bond, the energy of the ground state of the CHD cation (D_0_) increases (see Figure S12). At around ∼30 fs, the band loses intensity. Band B starts at ∼7.0 eV, but it is almost immediately stabilized to the 5.8–6.2 eV binding energy range. The sudden stabilization of band B is caused by the crossing of the cationic D_1_ and D_2_ states (see Figure S9). The maximum of the intensity of band B is reached at ∼40 fs. Figure 5b shows the average population of the adiabatic states S_0_, S_1_, and S_2_, as obtained from nonadiabatic dynamics simulations. By comparing the time evolution of bands A and B with the average population of the adiabatic states, one sees that the loss of intensity of band A coincides with the transfer of population from the S_1_ to the S_2_ state, with the maximum at ∼30 fs, while the maximum of the intensity of band B at ∼40 fs coincides with the rather counterintuitive rise of the population of the S_0_ state. The electronic populations of the diabatic states (Figure 5c) provide a more consistent view. The population initially residing in the 1^1^B state steadily decreases. The 2^1^A^–^ state is transiently populated at early times, but its population never exceeds 0.2. In the ∼35–55 fs interval, the population of the 1^1^A^–^ (blue) and 3^1^A^–^ (orange) states increases, and the system evolves in a superposition of these two states. This interval coincides with the maximum of the intensity of band B. The decomposition of the photoelectron spectra into the contributions from the diabatic states in Figure 5d–g unambiguously shows that the increase of the intensity of band B at around 40 fs originates from the population of the 3^1^A^–^ state (Figure 5g). As the reaction proceeds, the formation of the CHD and HT products correlating with the 1^1^A^–^ and 3^1^A^–^ states, respectively, becomes clearly visible.

Our analysis clearly points toward a predominant role of the 3^1^A^–^ diabatic state in the reactive path and, in general, that a more consistent picture of the overall dynamics can be achieved by analyzing diabatic rather than adiabatic contributions. We consider these findings as a step forward with respect to the description usually reported in the literature.^11−22^ We rely on both experimental advances, such as the possibility of measuring time-resolved valence photoelectron spectra with rather high electron energy resolution, and theoretical methods allowing accurate calculations of the photoelectron signal and on-the-fly adiabatic-to-diabatic transformation of electronic populations.

To draw a comprehensive picture of the ring-opening mechanism, we consider previously reported results and compare them to our results. From the experimental side, our time-resolved valence photoelectron spectra measured with high electron kinetic energy resolution do not contradict the previously reported results, such as, for example, those obtained with transient absorption spectroscopy^15^ and valence photoelectron spectroscopy with EUV pulses^20^ to quote previous works in which the probe was an electronic structure technique. The ring-opening reaction in CHD was previously modeled with a variety of computational approaches including reaction path calculations,^3,6,60−62^ wave packet simulations on reduced dimensionality PESs,^7,63−65^ full-dimensional ab initio multiple spawning,^19,66,67^ and surface hopping mixed quantum-classical dynamics.^9−13,20^ From SH simulations, it emerges that the dynamics proceeds dominantly on the lowest adiabatic surface, takes place on a sub-100 fs timescale, and leads to HT production with a yield slightly lower than 50%. This is in good agreement with our results. Larger differences are found with respect to density functional theory (DFT)-based studies of Schalk et al.^11^ in which a significantly larger quantum yield for HT production (64%) was obtained and of Filatov et al.^13^ in which a significantly longer decay constant (τ ∼ 235 fs) of the S_1_ state was predicted.

Concerning the photochemical mechanism, the commonly accepted mechanism—photoexcitation to the 1^1^B state, internal conversion to the 2^1^A^–^ state, and then CHD/HT bifurcation at 2^1^A^–^/1^1^A^–^ CI—was challenged by Schalk el al.^11^ The authors performed SH simulations in the S_1_ state using TDDFT, that is, with a method that cannot describe states with double excitation character, and nevertheless obtained HT with a high quantum yield. On these grounds, the authors proposed that an adiabatic description, in which a single electronic state gradually changes character along the reaction path, was adequate and that a doubly excited state was not necessarily involved in the ring-opening reaction. This assumption was not justified by the subsequent high-level XMS-CASPT2 simulations of Polyak et al.,^12^ where the authors reported that the double excitation character of 2^1^A^–^ contributes to the nature of the S_1_ and S_2_ states at the minimal energy S_1_/S_2_ CI as well as afterward. The recent combined experimental and theoretical study of Karashima and co-workers^20^ confirmed that two electronic states are involved in the reactions. They decomposed the two-dimensional maps of photoelectron spectra in contributions from two states, denoted S_1_* and S_1_**, but did not assign explicit diabatic labels to these states. However, as only two diabatic states, 1^1^B and 2^1^A^–^, were discussed in ref (20), the reader was left with the implicit assumption that the reactive S_1_** corresponds to the doubly excited 2^1^A^–^ state.

In this work, we have performed an adiabatic-to-diabatic transformation on top of surface hopping simulations in which seven diabatic states have been included. Our analysis revealed that the 3^1^A^–^ diabatic state plays a predominant role in the reaction and that the 2^1^A^–^ state is not the driving state but rather a spectator one. Namely, in contrast to the 2^1^A^–^ state, which exhibits no reduction of electron density on the breaking bond and consequently could hardy descend in energy by C_1_–C_6_ bond stretching, the 3^1^A^–^ state is a photochemically meaningful reactive state that can stabilize along the reaction path (compare Figures 2 and 3). We consider these findings as a step toward the full understanding of the ring-opening reaction in CHD and believe that the reinterpretation of the electronic character of the reactive state in CHD may also be of relevance for other pericyclic photoreactions.

## Conclusions

The photochemical ring-opening reaction of CHD to HT is a textbook example of a pericyclic reaction and possibly the most investigated isomerization reaction with advanced time-resolved spectroscopies. Here, we provide a new insight into the mechanism of the reaction. In particular, we show that the doubly excited dark state, labeled 2^1^A^–^, which is considered in the literature as the gateway to the isomerization process, does not play a significant role. Instead, an initially high-lying state, labeled 3^1^A^–^, with a pronounced double excitation character and a significant reduction of electron density upon the C_1_–C_6_ bond (the one which breaks during the isomerization process), is the reactive state whose temporal evolution drives the reaction.

## Methods

### Experimental Section

The experiments were performed at the LDM beamline^44^ at the FERMI FEL facility. The Ti/sapphire optical laser (pump) was operated at 267 nm, with a bandwidth of 1.2 nm. The FEL pulse (probe) was set at a photon energy of 19.23 eV, corresponding to the fourth harmonic of the seed wavelength of 258 nm. The spectrometer used to collect photoelectrons was a magnetic bottle.^45,46^ Electron time-of-flight (TOF) spectra were recorded shot-by-shot, while the delay between the pump and probe pulses was scanned with a step of 100 fs. The data used to construct Figure 1 consist of 15,000 shots per each delay, which were summed and normalized by the summed FEL intensity, recorded simultaneously for every shot. The electron flight times were converted to electron kinetic energies and calibrated according to ref (53) with respect to the FEL photon energy. The influence of the FEL and UV intensity on the TOF spectral shape was verified by performing a set of measurements at varied pulse energies and UV focus values. The third harmonic of the SLU at 267 nm has an estimated pulse duration of 120–170 fs, and the fourth harmonic of FERMI at 19.23 eV has an estimated pulse duration of ∼100 fs. A fwhm of 240 fs was used in the simulations, corresponding to a conservative evaluation of the overall time resolution. See Supporting Information for further details of experimental parameters, sample handling, and data analysis.

### Computational Section

In all calculations, CHD and HT were described by extended multistate complete active space self-consistent field second-order perturbation theory (XMS-CASPT2),^68,69^ employing an active space of six electrons in six orbitals, CAS[6e, 6o] (see Figure S7). In nonadiabatic dynamics simulations, the CASSCF orbitals were averaged over three states with equal weights. The three lowest electronic states of the CHD/HT cation (D_0_, D_1_, and D_2_) were taken into account in the computation of the photoelectron spectra. The cc-pVDZ basis set was used in all computations, and a real shift of 0.5 Hartree was employed to avoid intruder states in the dynamics. All electronic structure calculations were performed with the BAGEL program.^70,71^

The photoelectron spectra were computed using the classical limit of the doorway–window formalism.^72,73^ Nonadiabatic dynamics simulations were performed with Tully’s fewest switches surface hopping algorithm^47^ using an in-house code. The initial conditions were selected from the classical doorway function describing the excitation of the system by the pump pulse in our experiment.

Diabatic states were obtained from the adiabatic states by employing the diabatization scheme of Simah, Hartke, and Werner.^59^ To obtain smooth diabatic potentials, we included seven states in the calculations (XMS(7)-CASPT2[6e, 6o]). Details of the nonadiabatic dynamics simulations, the computation of photoelectron spectra, and the diabatization procedure are given in the Supporting Information.

## Supporting Information Available

Details regarding experimental setup and measurements on FERMI and the data analysis; details regarding the overall computational methodology, including static multireference calculations, nonadiabatic dynamics, spectrum calculation, and diabatization; and results that complement those presented in the article (PDF)

## Abbreviations

CHD: 1,3-cyclohexadiene
HT: 1,3,5-hexatriene
CoIn: conical intersection
FEL: free-electron laser
LDM: low density matter
ESA: excited-state absorption
FC: Franck–Condon
BAC*: extended bond-alternating coordinate
