# Supplementary material for: Photochemical Ring-Opening Reaction of 1,3-Cyclohexadiene: Identifying the True Reactive State
Source: J Am Chem Soc. 2022 Nov 29;144(48):21878–86. doi: 10.1021/jacs.2c06296 (PMC9732879; doi:10.1021/jacs.2c06296)
Supplement: Supplementary file 1 — ja2c06296_si_001.pdf [file ja2c06296_si_001.pdf]

Supporting information

for

The photochemical ring-opening reaction of 1,3-cyclohexadiene: identifying the true reactive state

Oksana Travnikova[1,‡], Tomislav Piteša[2,‡], Aurora Ponzi[2], Marin Sapunar[2], Richard James Squibb[3], Robert Richter[4], Paola Finetti[4], Michele Di Fraia[4], Alberto De Fanis[5], Nicola Mahne[6], Michele Manfredda[4], Vitali Zhaunerchyk[3], Tatiana Marchenko[1], Renaud Guillemin[1], Loic Journel[1], Kevin Charles Prince[4], Carlo Callegari[4], Marc Simon[1], Raimund Feifel[3], Piero Decleva[7], Nađa Došlić[2,*] and Maria Novella Piancastelli[1,8,*]

[1]*Sorbonne Université, CNRS, Laboratoire de Chimie Physique-Matière et Rayonnement, LCPMR, F-75005, Paris, France*

[2]*Institut Ruđer Bošković, Bijenička cesta 54, HR-10000 Zagreb, Croatia*

[3]*Department of Physics, University of Gothenburg, Origovägen 6B, SE-412 96 Gothenburg, Sweden*

[4]*Elettra-Sincrotrone Trieste, Strada Statale 14-km 163.5, 34149 Basovizza, Trieste, Italy*

[5]*European XFEL, D-22869 Schenefeld, Germany.*

[6]*IOM-CNR, S.S. 14 km 163.5 in Area Science Park, 34149 Trieste (Italy)*

[7]*Dipartimento di Scienze Chimiche e Farmaceutiche, Universitá di Trieste, I-34127 Trieste, Italy.*

[8]*Department of Physics and Astronomy, Uppsala University, SE-751 20 Uppsala, Sweden*

[‡] These authors have contributed equally.

*To whom correspondence should be addressed, maria-novella.piancastelli@physics.uu.se, nadja.doslic@irb.hr

Table of content

1. Experimental S3
- 1.1. Beam parameter S3
- 1.2. Spectrometer S3
- 1.3. Sample handling S4
- 1.4. Data analysis S4

2. Computational S6
- 2.1. Static calculations S6
- 2.2. Simulation of the time-resolved photoelectron spectrum S6
- 2.3. Reaction path calculations S9
- 2.4. Diabatization scheme S9

3. Supporting Tables S11

4. Supporting Figures S13

5. References S26

1. Experimental

1.1. Beam parameters

The beam profile of the pump laser, SLU, (wavelength: 266 nm, bandwidth 1.2 nm FWHM, duration 120-130 fs), was set to a circular spot of ~500 x 500 µm FWHM, so as to completely illuminate the profile of the FEL beam; the latter is adjusted by mechanically changing the shape of the focusing Kirkpatrick-Baez (K-B) mirrors.[1] At these long wavelengths, the divergence of the incoming FEL beam is large, and the vignetting due to the photon transport optics[1] results in a rectangular profile; the shape of the K-B was set for a profile of 300 µm x 300 µm. The polarization of both beams was set to horizontal.

The spatial and temporal overlap (t_0) of the FEL and SLU pulses were optimized by setting the FEL undulators to the 5th harmonic of the seed laser (257.85 nm), corresponding to 51.57 nm, i.e., the 1s$\rightarrow$5p resonance of the neutral He atom. The He+ ion yield of the resonant two-color two-photon (1+1') ionization was used as target signal for the manual optimization. The target spatial overlap is maintained over the course of the experiment with a precision of a few µm by means of an active feedback system, which tracks the position of a virtual beam on a commercial camera. Let us note that the pump and FEL pulses are transported along different paths, in a quasi-collinear arrangement.[2]

For the specific experimental conditions, the FEL harmonic was then set to the 4th harmonic of the same seed wavelength (19.23 eV), to avoid ionizing the helium carrier gas. Higher harmonics of the fundamental undulator resonance (in this case: the 8th and 12th harmonic of the seed) are normally present at the level of 0.1–1% and were abated by a suitable pressure of Ne in the gas attenuator that is part of the photon transport system[3]; let us note that Ne is transparent at the fundamental wavelength. Because the attenuator was then unavailable for its primary purpose, the FEL intensity was instead varied machine-side, via a combination of the strength of the seeding process and the number of active undulator modules. The pump laser is attenuated with a combination of rotatable and fixed polarizers. The pulse energy of the pump laser is measured with a commercial energy meter ((Gentech S-Link; Head QE8SP-B-BL-D0), and corrected to account for measured transport losses; the pulse energy of the FEL is measured with a gas energy meter,[4] and corrected for the calculated transport losses.[1]

1.2. Spectrometer

The electron kinetic energy spectra and photo-ion spectra were measured using a tandem electron-ion time-of flight spectrometer based on a modified magnetic bottle electron spectrometer (MBES).[5,6] The MBES operates as follows: by using a combination of a strong magnetic field located at the ionisation region, and a weak, homogenous magnetic field directed along the flight tube, photoelectrons which are initially emitted in all directions will have their trajectories parallelised towards the electron detector. The MBES therefore allows electrons to be collected over the full 4 π steradian solid angle and be detected with high efficiency (50-60% when microchannel plate efficiencies are included).
The weak magnetic field is generated by a solenoid with turn density 500/m, with a current of 0.3-2.0 ampère generating a magnetic field of the order of a few milliTesla. The solenoid starts ~ 90 mm from the interaction region and along the entire length of the ~2 m flight tube. A series of electrostatic lenses are positioned between the interaction region and the entrance to the flight tube in order to retard the electrons' kinetic energies, thus enhancing the

resolution. The strong magnetic field is produced by three NeFeB hollow cylindrical permanent magnets with a conical soft iron polecap used to concentrate the magnetic field. The magnet assembly produces a peak field strength of ~ 0.1 Tesla, and yields a dE/E of approx. 1/20 – 1/30.
Additionally, the hollow profile of the magnet assembly allows ions that are produced by the FEL to be accelerated through the magnet by two additional electrostatic lenses and subsequently a flight tube, with a total interaction region to detector distance of 200-205 mm and a total ion acceleration of 400 V to 5 kV depending on the experimental conditions. The electrons and ions are each detected by two Hamamatsu F9892-31 chevron microchannel plate assemblies located at the far ends of the two flight tubes. The electron and ion signals are sent to separate channels of a CAEN analogue to digital system and the full waveforms stored for later analysis.
The spectrometer can be operated in either a pulsed mode or a static mode whereby low, DC voltages are used to extract and accelerate the electrons. This also causes the electrons to be accelerated, however the lenses located just before the electron flight tube can be used to retard the electrons to their original kinetic energy or less to maintain the electron resolution. For higher masses, where higher extraction fields are required, the voltage applied between the magnet and the first electron lens element is initially kept at close to ground to allow the electrons to escape the interaction region. After a 1-200 ns delay, a Behlke GHTS solid-state switch is used to apply a voltage to extract the ions.

1.3. Sample handling

The sample was mixed with 1.8 bar He in an external stainless steel bubbler, and injected into the experimental chamber via a commercial pulsed valve (Parker, Model 9, nominal aperture diameter: 800 µm). The bubbler was kept in a temperature-controlled refrigerated bath, and the partial pressure of the sample molecule was determined by the bath temperature under the assumption of thermodynamic equilibrium; the literature values used were taken from [7]. The valve was operated at the repetition rate of the FEL (50 Hz); the relative delay between the valve opening and the FEL pulse was scanned to determine sample-pulse length (~250 µs FWHM, depending on the nominal opening time) and optimal synchronization conditions. If desired, one out of n shots can be a blank shot (specifically: one for which the sample valve is fired out-of-sync with the FEL), and the measurement subtracted from the regular shots, after proper scaling. This is advantageous to assess the contribution of residual gas in the chamber; where applicable, we report the number n=6 as "background period". Changing the delay of a blank shot is preferable to suppressing it altogether, to preserve the thermal stability of the valve.

1.4. Data analysis

Photoelectron time-of-flight (TOF) traces were measured shot-by-shot for different delay times between pump and probe pulses. The FEL pulse energy was recorded for every shot and its distribution is shown in Fig. S1. The shots with very low (below 3 µJ) and high (above 17 µJ) FEL intensity were excluded from the data analysis. The TOF traces were summed and normalised to the summed FEL pulse intensity after subtraction of the «blank» background shots. Then the photoelectron flight times were transformed to electron kinetic energies by a non-linear conversion, which was derived by correlation of the peak maxima to the literature values from Ref. [8] subtracted from the FEL photon energy (19.23 eV). The electron kinetic energies scale was then converted to the binding energy scale. In total 15000 shots were recorded per each delay point for the pump-probe delay time scans, which were recorded with

the step of 100 fs. Photoelectron spectra recorded for the time delay t_0, corresponding to the overlap of the UV-pump and FEL-probe pulses, UV pulse only and FEL pulse only are shown in Fig.S2. Evolution of the photoelectron spectra as function of the pump-probe delay is presented in Fig. S3 without subtraction of the ground-state spectrum. In Fig. 1 of the main manuscript, the ground-state spectrum was subtracted without adjustment of the spectral intensities. For this, the spectrum recorded at the negative delay time t=-0.7 ps was subtracted from all the photoemission spectra recorded at different delays. At t=-0.7 ps the FEL pulse arrives before the UV pulse, therefore only the ground-state CHD contributes to the photoemission.

Reference photoelectron spectra with only the FEL or UV pulse were recorded by accumulating in total 30000 shots per spectrum and a photoelectron spectrum when the FEL and UV pulses are overlapped at t_0 (pump-probe delay time t=0) was recorded by accumulating about 50000 shots. The zoom of these reference spectra is presented in Figure S3 for the electron binding energy region of interest.

An additional analysis was performed to check the quality of accumulated datasets, where the spread of the signal intensity during long pump-probe delay scans was plotted for each delay point. To do this, the traces were normalised to the FEL pulse intensity on shot-by-shot basis, then binned by 100 and the intensities, integrated over the specified TOF range, were histogrammed for each pump-probe delay point. The TOF regions, selected for this analysis, correspond to the binding energy regions, where the changes in the course of the ring-opening reaction of CHD are observed. The dataset with the least spread of the signal intensities was used for the constructions of Fig. 1 of the main manuscript and Fig. S4. The median distibutions of the intensities, integrated over the TOF regions of interest, were obtained as the center of gravity of the plotted histograms. These median distributions are qualitativitly similar to the integrated areas of the 2D photoelectron map presented in Fig. 1c of the main paper and allow tracking evolution of the photoelectron signal as a function of the pump-probe delay.

2. Computational

2.1. Static calculations

All electronic structure calculations were performed using extended multi-state (XMS) complete active space self-consistent field second-order perturbation (XMS-CASPT2) theory [1–3].[9–11] For the neutral CHD an active space of 6 electrons in 6 orbitals was used (CAS(6,6)), while an active space of 5 electrons in 6 orbitals (CAS(5,6)) was used for the cation. The orbitals constituting the active space computed at the equilibrium geometry of CHD are shown in the Fig. S6. Nonadiabatic dynamics simulations were carried out in the manifold of the three lowest electronic states (S_0, S_1, S_2) of neutral CHD (XMS(3)-CASPT2(6,6)). A real shift of 0.5 Hartree was used to avoid intruder states in the dynamics. To compute the photoionization spectrum the three lowest electronic states of the CHD cation (D_0, D_1, D_2) were taken into account. The cc-pVDZ basis set was employed in all computations.
For the computation of the diabatic states along the ring opening reaction path and along nonadiabatic trajectories, state averaging with equal weights was performed over 7 states (XMS(7)-CASPT2(6,6)). All electronic-structure calculations were performed with the BAGEL program.[12,13]

2.2. Simulation of the time-resolved photoelectron spectrum

Theoretical approach

The time-resolved photoelectron spectrum was computed in the classical limit of the doorway-window (DW) approximation.[14–17] A detailed description of the application of the classical DW formalism for the computation of time-resolved photoelectron spectra is given in Ref. 17. Briefly, the computational procedure includes: (i) the description of the excitation of the system by the pump pulse, (ii) the propagation of classical trajectories in the electronic ground and excited states and (iii) the description of the photoionization by the probe pulse.

(i) The pump pulse centered at $t = 0$, with carrier frequency ω_{pu} is assumed to have a Gaussian shape with envelopes $\varepsilon_{pu}(t)$ and $\tilde{\varepsilon}_{pu}(\omega)$ in the time and energy domain, respectively. A set of initial geometries **R** and momenta **P**, as well as the initial excited state are stochastically sampled from the classical doorway function[16,17]

$$D_I(\mathbf{R},\mathbf{P};\omega_{\mathrm{pu}}) = \tilde{\varepsilon}^2_{\mathrm{pu}}\left(\omega_{\mathrm{pu}} - \Delta E_{IG}(\mathbf{R})\right)|\mu_{GI}|^2\rho^{\mathrm{Wig}}(\mathbf{R},\mathbf{P}), \quad (1)$$

where $\Delta E_{IG}(\mathbf{R}) = E_I(\mathbf{R}) - E_G(\mathbf{R})$ is the vertical excitation energy between the ground state G and excited electronic state I, $\mu_{GI}(\mathbf{R})$ is transition dipole moment and $\rho^{\mathrm{Wig}}(\mathbf{R},\mathbf{P})$ is the ground-state Wigner distribution.

(ii) Starting with the sampled initial conditions, surface hopping trajectories were propagated in the excited electronic states. To describe the “hole” in the ground electronic state, Born–Oppenheimer molecular dynamics simulations were performed with the same initial conditions.

(iii) The action of the probe in the excited states at the delay time τ is described by the classical window function

$$W_{\mathrm{I}}\left(E_k,\tau;\omega_{\mathrm{pr}}\right)=\sum_F \tilde{\varepsilon}_{\mathrm{pr}}^2\left(\omega_{\mathrm{pr}}-E_k-IE_{I(\tau)F}(\tau)\right)\sigma_{I(T)F}(E_k,\tau), \quad (2)$$

where $\tilde{\varepsilon}_{pr}(\omega)$ denotes the Fourier transform of the probe pulse envelope $\varepsilon_{pr}(t)$ and E_k the kinetic energy of the photoelectron. $IE_{I(\tau)F}(\tau)=IE_{I(\tau)F}(\mathbf{R}(\tau))$ is the ionization energy of the initial state $I=I(\tau)$ to the final state F and $\sigma_{I(\tau)F}(E_k,\tau)=\sigma_{I(\tau)F}(E_k,\mathbf{R}(\tau))$ is the partial cross section. Similarly, the window function

$$W_0\left(E_k,\tau;\omega_{\mathrm{pr}}\right)=\sum_F \tilde{\varepsilon}_{\mathrm{pr}}^2\left(\omega_{\mathrm{pr}}-E_k-IE_{GF}(\tau)\right)\sigma_{GF}(E_k,\tau), \quad (3)$$

detects the "hole" in the ground state. Here $IE_{GF}(\tau)$ and $\sigma_{GF}(E_k,\tau)$ are coordinate dependent ionization energies and partial cross sections for the ionization from the ground state G.

The time-resolved photoelectron spectrum $P(E_k,\tau)$ is obtained by averaging over all trajectories as

$$P(E_k,\tau)=\langle D_I(\mathbf{R},\mathbf{P};\omega_{\mathrm{pu}})W_{\mathrm{I}}\left(E_k,\tau;\omega_{\mathrm{pr}}\right)\rangle-\sum_I\langle D_I(\mathbf{R},\mathbf{P};\omega_{\mathrm{pu}})W_0\left(E_k,\tau;\omega_{\mathrm{pr}}\right)\rangle. \quad (4)$$

The first term corresponds to excited state absorption (ESA) component and the second to the ground state bleach (GSB) components of the spectrum. Finally, the time-resolved photoelectron spectrum $P(E_k,\tau)$ is expressed in terms of the binding energy, $\mathrm{eBE}=\omega_{\mathrm{pr}}-E_k$.

Computational protocol and details

We sampled stochastically a first set of 2000 coordinates and momenta from the ground-state Wigner distribution $\rho^{\mathrm{Wig}}(\mathbf{R}_{\mathrm{G}},\mathbf{P}_{\mathrm{G}})$. By accounting for the shape of the pump pulse and the oscillator strength of the transition, $\tilde{\varepsilon}_{\mathrm{pu}}^2|\mu_{GI}|^2$, we obtained a set N = 107 initial coordinates **R** and momenta **P**, as well as a set of initial excited states I. In our experiment the pump pulse is characterized by $\delta_{pu}=240$ fs FWHM in the time domain and $\tilde{\delta}_{pu}=7$ meV FWHM in the energy domain. Owing to the narrowness of the pump in the energy domain, we replaced $\tilde{\varepsilon}_{\mathrm{pu}}^2|\mu_{GI}|^2$ with the rectangular function $\Pi(\omega_{pu}\pm 5\tilde{\delta}_{pu})$ to increase the efficiency of the sampling.

To compute the ESA component of the photoionization spectrum the trajectories were propagated in the excited states with Tully's fewest switches surface hopping (FSSH) algorithm[18] using an in-house code.[19,20] Newton's equations for nuclear motion were propagated with the velocity-Verlet algorithm for 2000 fs. The usual time step of 0.5 fs was used. The locally diabatic formalism was used to propagate the electronic wave function and compute the hopping probabilities.[21] The energy-based decoherence procedure of Granucci and Persico[22] was employed (α = 0.1 Hartree). In the simulations 106 out of 107 trajectories were started in the first excited state (S_1) and one trajectory was started from the second excited state (S_2). In CAS-based nonadiabatic dynamics simulations the total energy is not always well conserved. Indeed, we found that in 4 out 107 trajectories the standard deviation of the total energy was larger than 0.2 eV, while in 14 trajectories the deviation was between

0.05 and 0.2 eV. Since the fluctuation of the total energy had little effect on the averaged adiabatic and diabatic state populations, we have included these trajectories in the simulations of the photoelectron spectrum.

At the end of the surface hopping simulations we evaluated the window function $W_\mathrm{I}(E_k,\tau;\omega_\mathrm{pr})$ and obtained the ESA signal by averaging over all trajectories. For the GSB component of the spectrum we propagated the sampled trajectories in the electronic ground state using the same velocity-Verlet algorithm and the 0.5 fs time step. We then evaluated $W_0(E_k,\tau;\omega_\mathrm{pr})$ and averaged over all trajectories to obatain the GSB signal.

In our simulations the partial cross sections $\sigma_{I(\tau)F}$ and σ_{GF} needed for the evaluation of the windows functions were approximated with Dyson orbital norms *(vide infra*) and the experimental time-resolution (Fig. 1 and Fig. S5) was accounted for by convoluting the spectrum (Eq. 4) with a Gaussian pump-probe cross-correlation function[23] of 328 fs FWHM.

Dyson orbitals

To simulate the time-resolved photoelectron spectrum the partial differential cross section needs to be computed

$$\frac{d\sigma_{IFk}}{d\vec{k}} = 4\pi^2\alpha\omega|\langle\Psi_I^N|\vec{\mu}|\Phi_{Fk}^N\rangle|^2 \tag{5}$$

where $\vec{k}$ is the momentum of the photoelectron, α is the fine structure constant, ω is the photon energy and $\langle\Psi_I^N|\vec{\mu}|\Phi_{Fk}^N\rangle = \vec{\mu}_{IFk}$ is the dipole transition moment computed for a particular molecular geometry from the initial electronic state I and the final state F. Expressing the wave function of the final state, Φ_{Fk}^N, as an antisymmetrized product of the wave function of the ejected photoelectron, φ_k and the bound cationic wave function Ψ_F^{N-1} one obtains

$$\vec{\mu}_{IFk} = \langle\Psi_I^N|\vec{\mu}|\Phi_{Fk}^N\rangle = \langle\phi_{IF}^D|\vec{\mu}|\varphi_k\rangle \tag{6}$$

where ϕ_{IF}^D is the Dyson orbital describing the hole created in Ψ_F^{N-1}, given as

$$\phi_{IF}^D = \sum_k \langle\Psi_F^{N-1}|\hat{a}_k|\Psi_I^N\rangle\chi_k \tag{7}$$

and $\hat{a}_k$ is the annihilation operator for the molecular orbital χ_k. The procedure for the computation of Dyson orbitals from CASPT2 wave function overlaps is given in Ref. 17.

In order to simulate the time-resolved photoelectron spectrum many hundreds of evaluations of the cross section are needed. Even for medium size systems as CHD accurate computations of the continuum wave functions are numerically expensive and approximations are usually done. In the sudden approximation in which the photoelectron and the cation are decoupled, the spectral strength of a transition

$$|\vec{\mu}_{IFk}|^2 = |\langle \phi_{IF}^D|\vec{\mu}|\varphi_k\rangle|^2 = \|\phi_{IF}^D\|^2 \left|\langle \bar{\phi}_{IF}^D|\mu|\varphi_k\rangle\right|^2 \approx \|\phi_{IF}^D\|^2 \quad (8)$$

is given by the square of the norm of the Dyson orbital, $\|\phi_{IF}^D\|^2$, where $\bar{\phi}_{IF}^D = \phi_{IF}^D/\|\phi_{IF}^D\|$ is the Dyson orbital normalized to one. Since in our experiment delocalized valence electrons are ejected by a high-energy probe pulse, the sudden approximation holds well.

2.3. Reaction path calculations

A reaction path in C_2 symmetry was constructed by linear interpolation in internal coordinates between the minimum of the first state of symmetry A, that is the Franck-Condon geometry (1A), and the minimum of the second state of the same symmetry, 2A. The two minima have been optimized using Molpro[24] in C_2 symmetry. In addition to the 12 geometry points connecting the two minima, 3 geometries were extrapolated on each side of the minima resulting in a total of 19 geometries on the reaction path. The geometries were rotated to fulfil the Eckart conditions with respect to the Franck-Condon geometry.

2.4. Diabatization scheme

The recent review by Shu *et al.*[25] provides a comprehensive overview of different strategies for calculating diabatic states of molecules. Notwithstanding recent progresses, the calculation of diabatic populations in nonadiabatic surface-hopping simulations remains a complicated task.[26] Herein we employed the diabatization scheme presented by Simah, Hartke, and Werner[27] for CASSCF states, but trivially extended it for XMS-CASPT2 states (*vide infra*). The idea behind this approach is to obtain not necessarily quasidiabatic states, i.e. the ones with minimized nuclear-momentum couplings, but rather so-called configurationally-uniformed states, i.e. the ones whose electronic characters change smoothly with molecular geometry (check second part of section III.C. in Ref. 25). More precisely, we choose the diabatic states to be equal to the adiabatic states on the Frack-Condon geometry, $\mathbf{R}_{\mathrm{FC}}$. On some displaced geometry **R**, the diabatic states were then obtained by an unitary transformation of the adiabatic states on **R**, i.e. as $\boldsymbol{\Psi}^{(\mathrm{d})} = \mathbf{T}\boldsymbol{\Psi}^{(\mathrm{a})}$, where the adiabatic-to-diabatic transformation matrix **T** is obtained by symmetric orthogonalization of the overlap matrix between chosen electronic states on $\mathbf{R}_{\mathrm{FC}}$ and **R**. To obtain the configurationally-uniformed states, the electronic wave-function overlaps were calculated as a dot product of the CI vectors transformed to the basis of so-called diabatic Slater determinants containing only diabatic active orbitals. The diabatic orbitals at $\mathbf{R}_{\mathrm{FC}}$ are identical to active adiabatic orbitals, while at **R** they are obtained as symmetrically-orthogonalized projections of active adiabatic orbitals on **R** to the diabatic active orbitals. The CI vectors in the basis of adiabatic Slater determinants were previously obtained by rotation of SA-CASSCF CI vectors with the XMS-CASPT2 rotation matrix.[28]

In the reaction-path calculations, we included seven electronic states. From the total overlap matrix with the reference geometry (7×7) we extracted submatrices of dimension $n\times n$, which were orthogonalized and used as the adiabatic-to-diabatic transformation matrix **T**. The diabatic potentials, which are the diagonal elements of the diabatic Hamiltonian, were obtained by unitary transformation of the adiabatic Hamiltonian, $\mathbf{H}^{(\mathrm{d})} = \mathbf{T}\mathbf{H}^{(\mathrm{a})}\mathbf{T}^T$. Smooth diabatic potentials were obtained with $n = 6$ for smaller C_1-C_6 distances (< 1.9 Å) and with $n = 5$ for larger C_1-C_6 distances (see Fig. 3).

In nonadiabatic dynamics simulations, our goal was to find the diabatic basis that best spans the active (currently populated) state of each surface-hopping trajectory at each geometry. In this way the diabatic character of the active adiabatic state, mostly the S_1 state, could be retrieved. For most geometries, the active state could be spanned in the basis of 4 diabatic states (1^1A^-, 1^1B, 2^1A^- and 3^1A^-). Therefore, along each surface-hopping trajectory we performed XMS(7)-CASPT2(6,6) single point calculations in steps of 2 fs, computed the 7×7 wave-function overlap matrix with the reference geometry, and extracted the 4×4 submatrix containing the four diabatic states of interest, the active adiabatic state and three other adiabatic states which maximize the Frobenius norm of the extracted matrix. The average population of a diabatic state, a, computed for a swarm of $i = 1,2,\ldots,N$ nonadiabatic trajectories running in the adiabatic surface, $I(\tau)$, is then given as[17,29]

$$P_a(\tau) = \frac{1}{N}\sum_i^N \left|T_{aI_i(\tau)}(\mathbf{R}_i(\tau))\right|^2.$$

The contribution from the ionization of the a-th diabatic state in the ESA component of the photoionization spectrum was calculated as

$$P_a^{(\mathrm{diab})} = \langle D_I\big(\mathbf{R},\mathbf{P};\omega_{\mathrm{pu}}\big)W_a^{(\mathrm{d})}\big(E_k,\tau;\omega_{\mathrm{pr}}\big)\rangle, \tag{9}$$

where $W_a^{(\mathrm{d})}$ is the window function of the a-th diabatic state

$$W_a^{(\mathrm{d})}\big(E_k,\tau;\omega_{\mathrm{pr}}\big) = \left|T_{aI(\tau)}\big(\mathbf{R}(\tau)\big)\right|^2 W_{\mathrm{I}}\big(E_k,\tau;\omega_{\mathrm{pr}}\big), \tag{10}$$

and $T_{aI(\tau)}(\tau)$ is a matrix element of adiabatic-to-diabatic transformation matrix connecting the active state $I(\tau)$ and the a-th diabatic state.

3. Supporting Tables

Table S1. State ordering and vertical excitation energies of the lowest 6 excited states of A symmetry and lowest 4 excited states of B symmetry calculated with linear response (LR) coupled cluster singles, doubles, and triples model CC3 and the cc-pVDZ basis set at the Franck-Condon geometry of CHD. On the basis of the CI coefficients of the leading CSFs, one sees that the 2A, 6A and 7A states on LR-CC3 level correspond to the 2^1A^-, 1^1A^+ and 3^1A^- on XMS-CASPT2 level respectively.

State	E_{exc} / eV (LR-CC3)	Leading configurations	CI coefficients
1B	5.45	$\pi_2 \rightarrow \pi_1^*$	0.94
2A	6.54	$\pi_2\pi_2 \rightarrow \pi_1^*\pi_1^*$<br>$\pi_1 \rightarrow \pi_1^*$<br>$\pi_2 \rightarrow \mathrm{Ry}(3\mathrm{p}_z)$<br>$\pi_2 \rightarrow \pi_2^*$	0.46<br>–0.41<br>–0.34<br>0.22
3A	7.26	$\pi_2 \rightarrow \mathrm{Ry}(3\mathrm{s})$	0.90
2B	7.29	$\sigma_2 \rightarrow \pi_1^*$	0.91
4A	7.71	$\sigma_1 \rightarrow \pi_1^*$	0.92
5A	8.19	$\pi_2 \rightarrow \mathrm{Ry}(3\mathrm{p}_z)$<br>$\pi_1 \rightarrow \pi_1^*$<br>$\pi_2 \rightarrow \mathrm{Ry}(3\mathrm{s})$<br>$\pi_2\pi_2 \rightarrow \pi_1^*\pi_1^*$	0.84<br>0.29<br>0.20<br>0.18
3B	8.34	$\pi_2 \rightarrow \mathrm{Ry}(3\mathrm{p}_x)$<br>$\pi_1 \rightarrow \mathrm{Ry}(3\mathrm{s})$	0.90<br>0.23
6A	8.82	$\pi_1 \rightarrow \pi_1^*$<br>$\pi_2 \rightarrow \pi_2^*$<br>$\pi_2\pi_2 \rightarrow \pi_1^*\pi_1^*$	0.53<br>0.67<br>0.29
4B	8.94	$\pi_2 \rightarrow \mathrm{Ry}(3\mathrm{p}_y)$	0.90
7A	9.09	$\pi_2\pi_2 \rightarrow \pi_1^*\pi_1^*$<br>$\pi_2 \rightarrow \pi_2^*$<br>$\pi_1 \rightarrow \pi_1^*$	0.44<br>–0.56<br>0.35

4. Supporting Figures

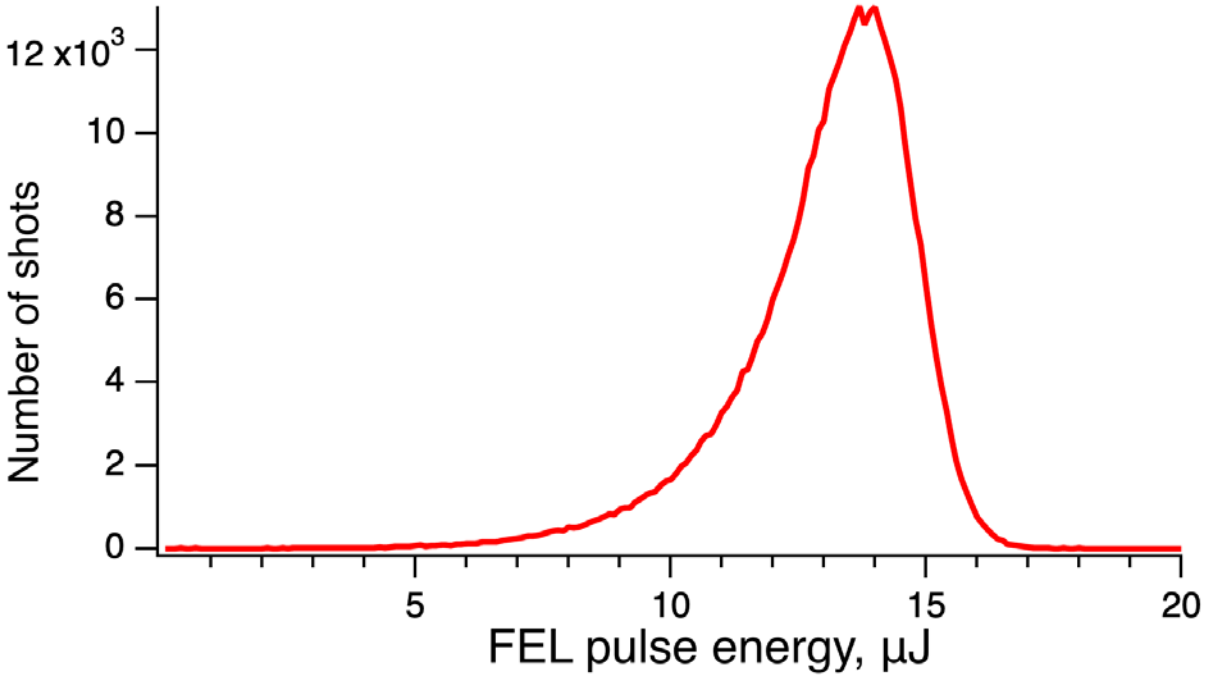

Figure S1. Distribution of the FEL pulse energy for about 400000 shots during one pump-probe delay scan measurement. The data are sampled with increments of 0.1 µJ.

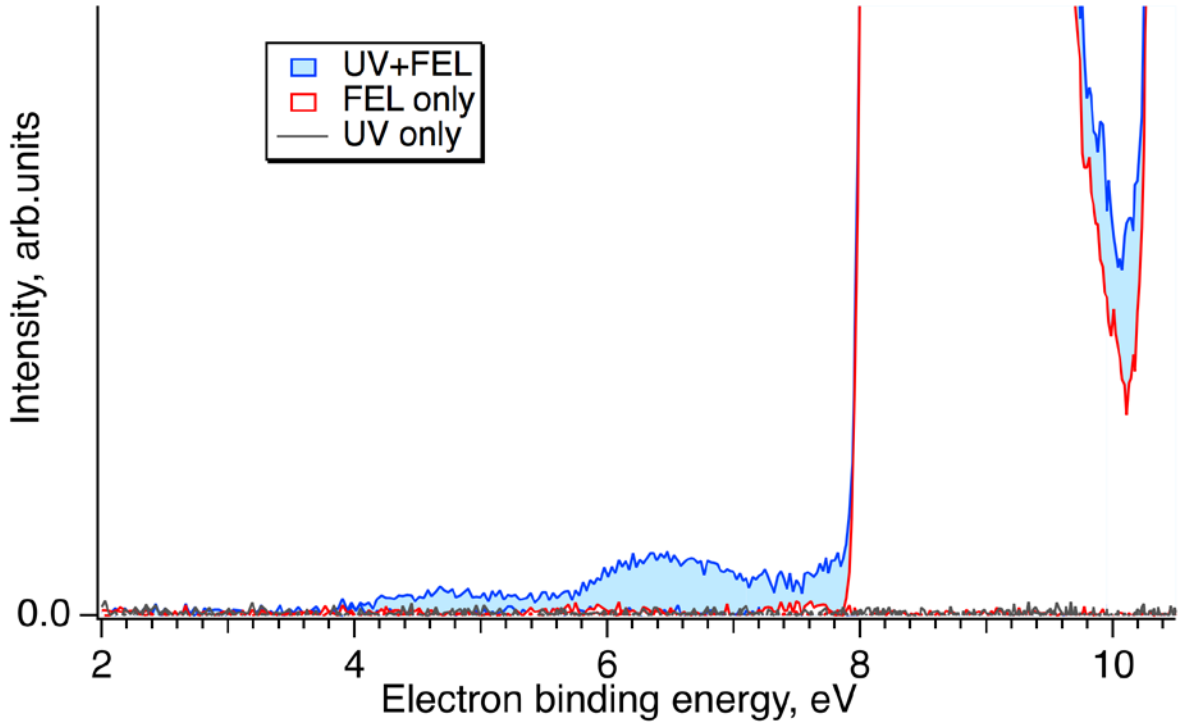

Figure S2. Photoelectron spectra recorded for (1) the time delay t_0, corresponding to the overlap of the UV-pump and FEL-probe pulses – blue curve; (2) UV pulse only – grey curve and (3) FEL pulse only – red curve.

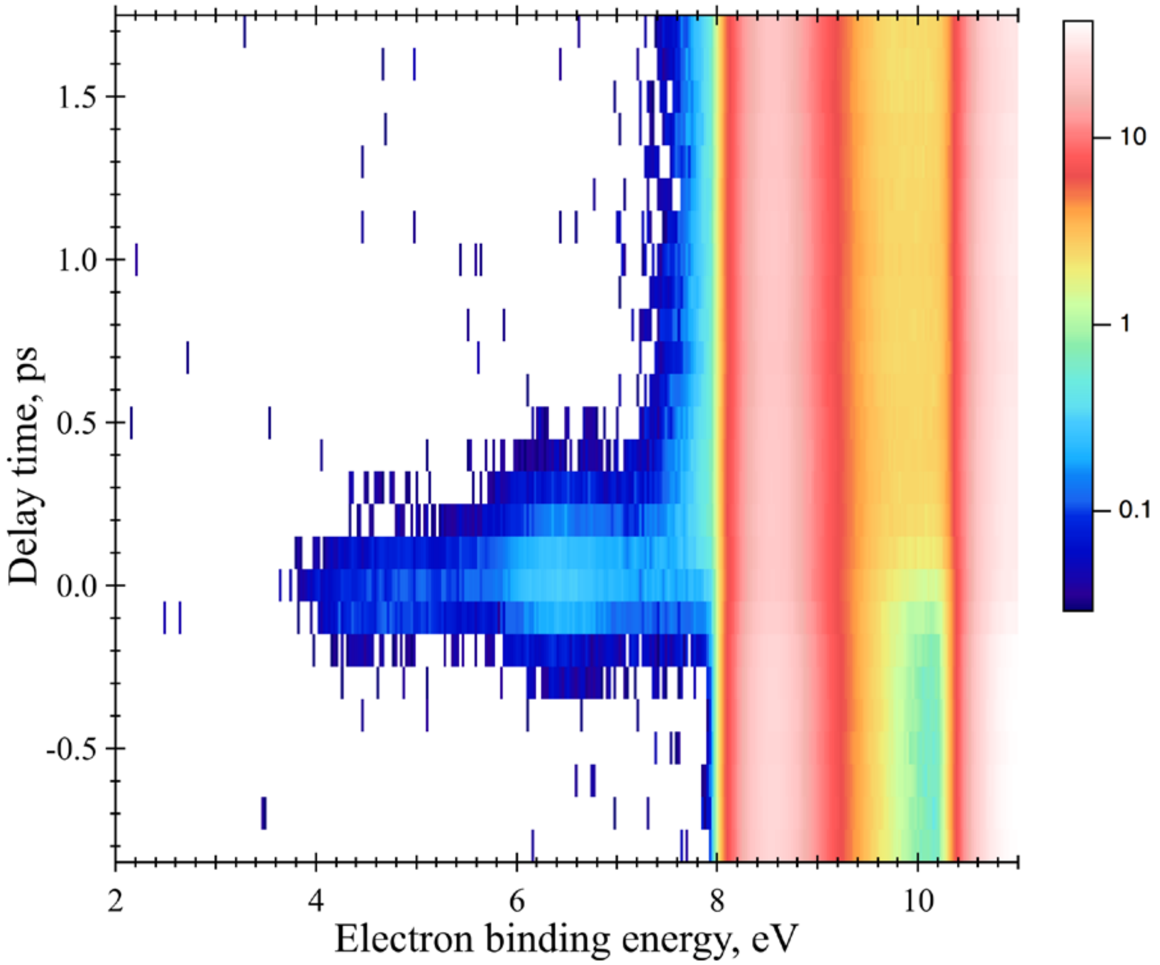

Figure S3. Evolution of the photoelectron spectra as a function of the pump-probe delay, measured with a step of 100 fs. The 2D map shown here is without subtraction of the ground-state spectrum and was used to construct Figure 1 of the main paper.

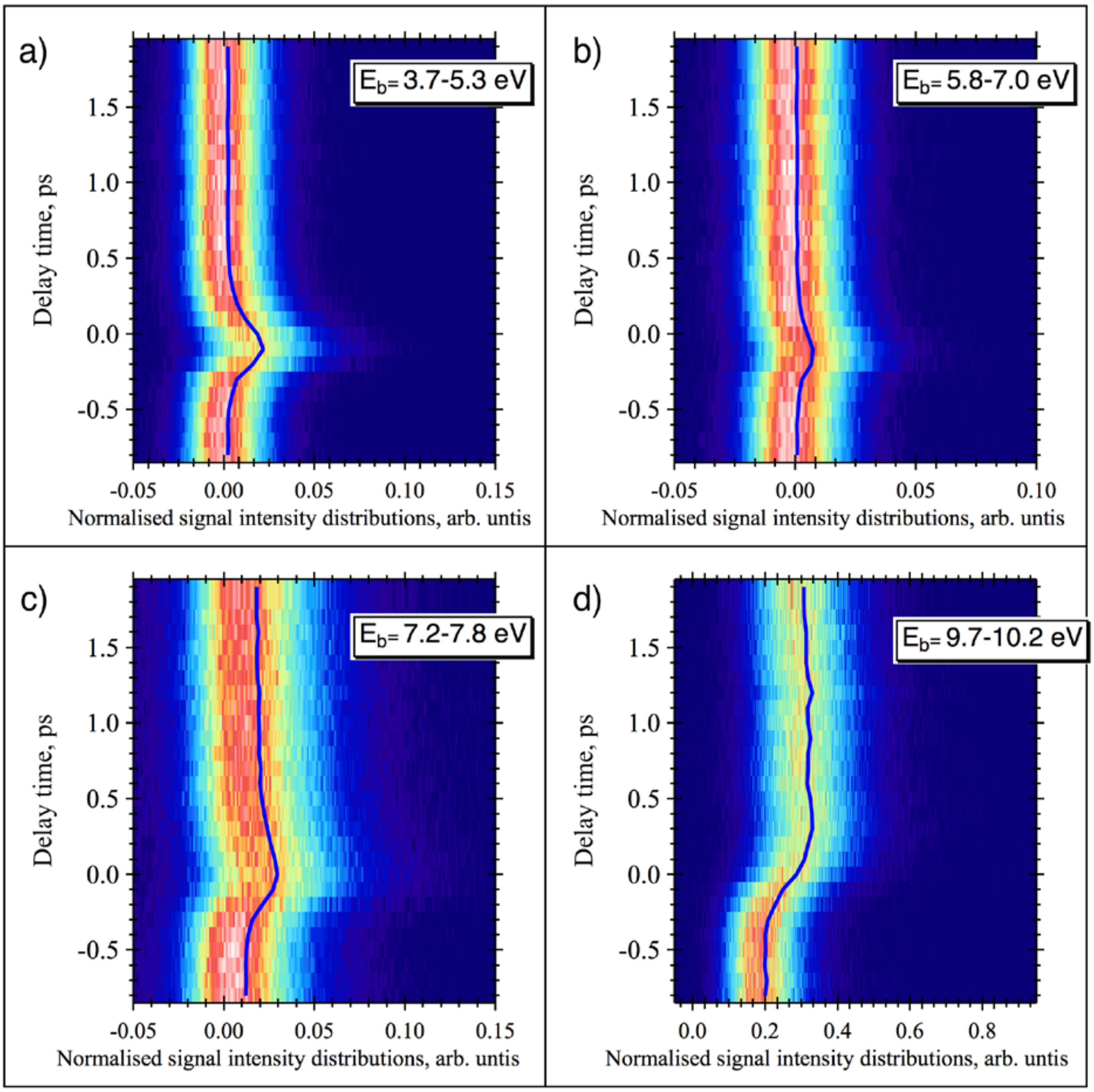

Figure S4. Normalised time-of-flight signal intensity distributions, integrated over the photoelectron TOF regions, which correspond to the photoelectron binding energy regions of about a) 3.7-5.3 eV, b) 5.8-7.0 eV, c) 7.2-7.8 eV and d) 9.7-10.2 eV. The histograms of the intensity distributions were obtained for each delay point of the pump-probe delay scan of one dataset. The blue curves correspond to the median intensity distributions and show the evolution of the photoelectron signal as a function of the pump-probe delay time.

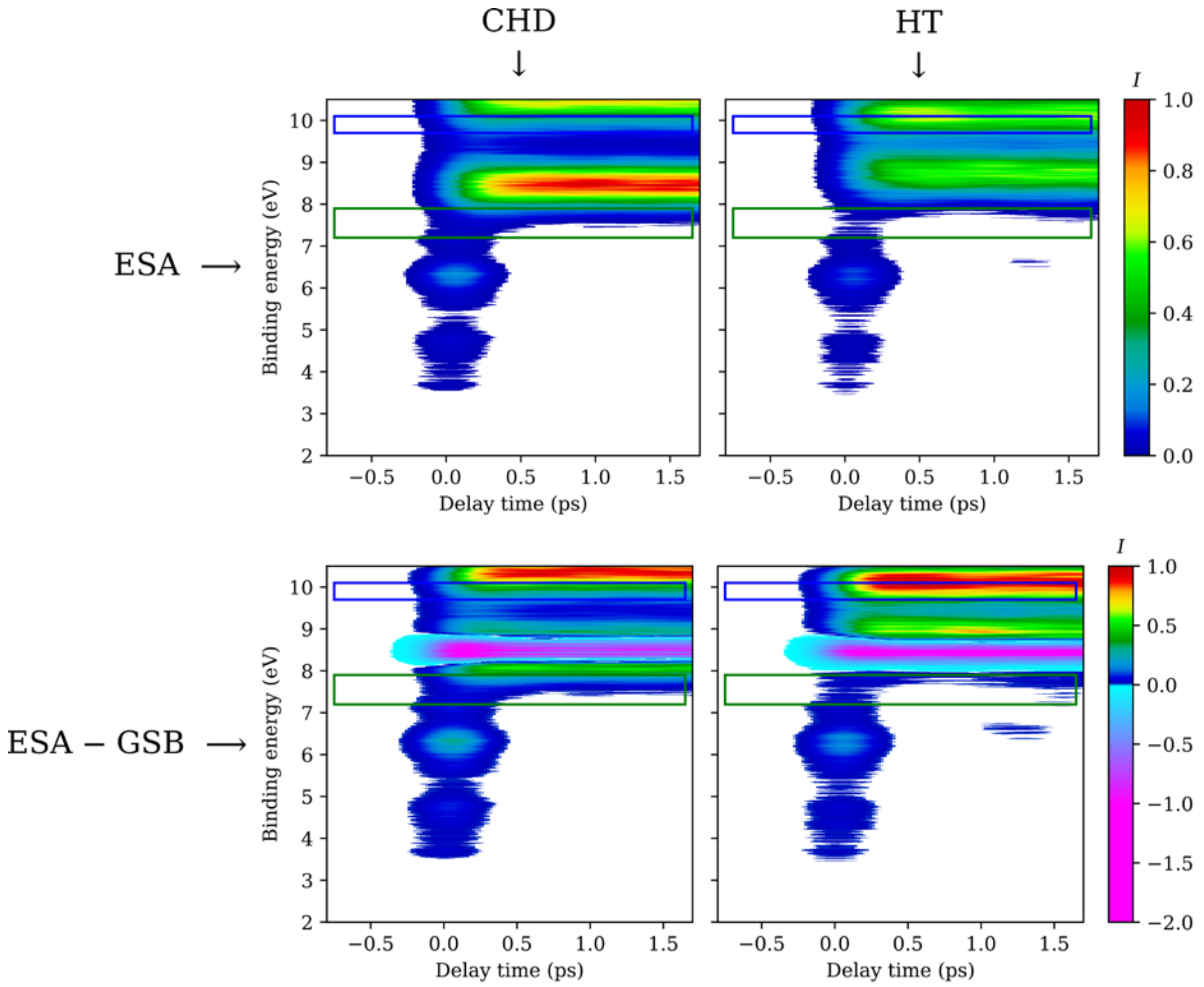

Figure S5. Upper panels: Simulated time-resolved photoelectron spectra of pump-excited geometries propagated in the excited states using classical trajectory surface hopping dynamics and ending as CHD (left) or HT (right). The spectrum is computed as the excited-state absorption (ESA) component of the transient absorption pump-probe signal for excited states in the continuum (for details, see SI sec. 2.2) Lower panels: Time-resolved photoelectron spectra obtained by subtracting the ground-state bleach (GSB) contribution from the ESA contribution. The GSB contribution is computed by propagating the pump-excited geometries in the electronic ground state. Bands C and D are marked with green and blue rectangles, respectively.

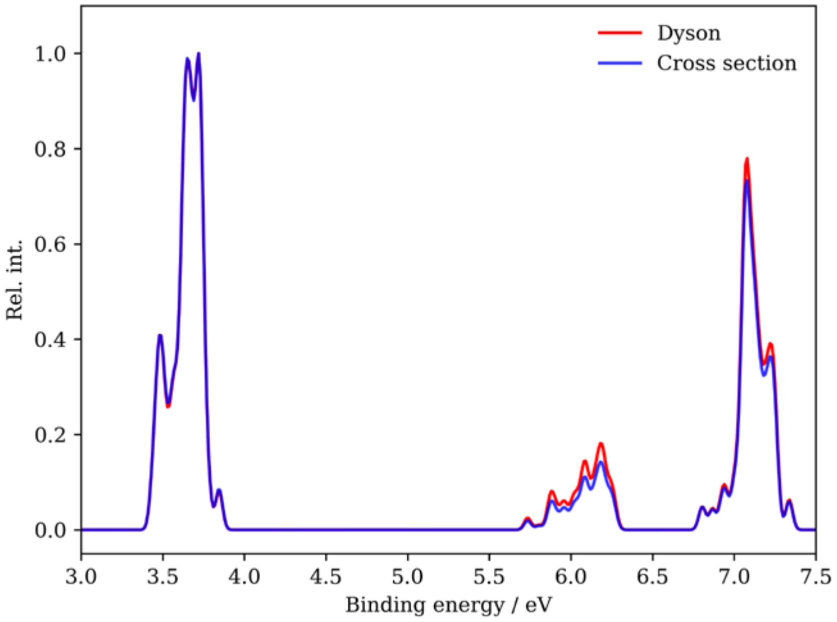

Figure S6. Comparison between photoelectron spectra of CHD calculated using square norms of Dyson orbitals (red) and partial cross sections (blue). The calculations were performed for 46 randomly selected geometries from the initial set of N_{traj} = 107 geometries of CHD. The line signals were broadened by a Gaussian function with FWHM = 0.05 eV.

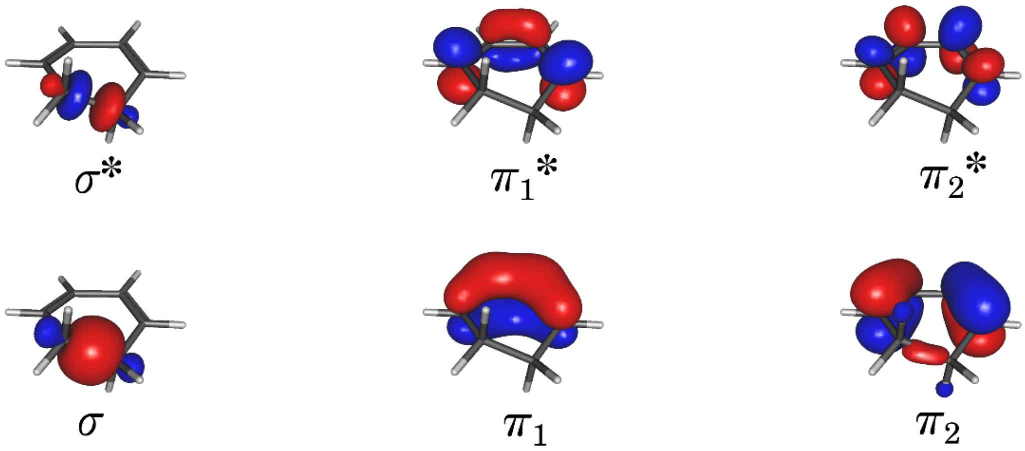

Figure S7. The orbitals constituting the (6e,6o) active space at the ground state minimum energy geometry of CHD (C_2 symmetry).

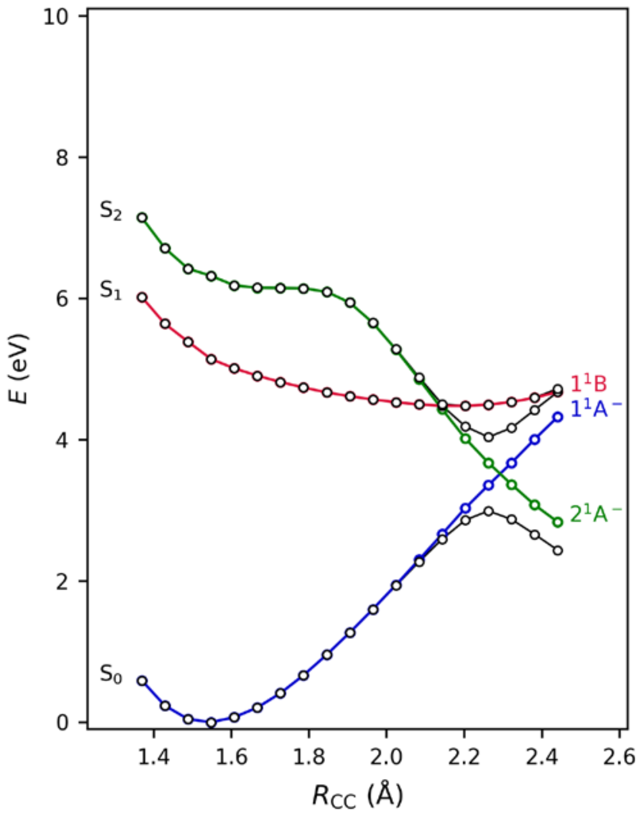

Figure S8. Coordinate dependence of the potential energy curves of the adiabatic (black) and diabatic (colours) electronic states obtained with XMS(3)-CASPT2. The diabatization includes the S_0-S_2 adiabatic and 1^1A^-, 1^1B and 2^1A^- diabatic states. Because the number of states included in the adiabatic-to-diabatic transformation is too small, the 2^1A^- state (green) apparently crosses with the 1^1A^- state (blue). Details of the diabatization procedure are given in Sec 2.4. For a comparison of adiabatic potential energy profiles calculated with XMS(3)-CASPT2 and XMS(7)-CASPT2 see Figure S9.

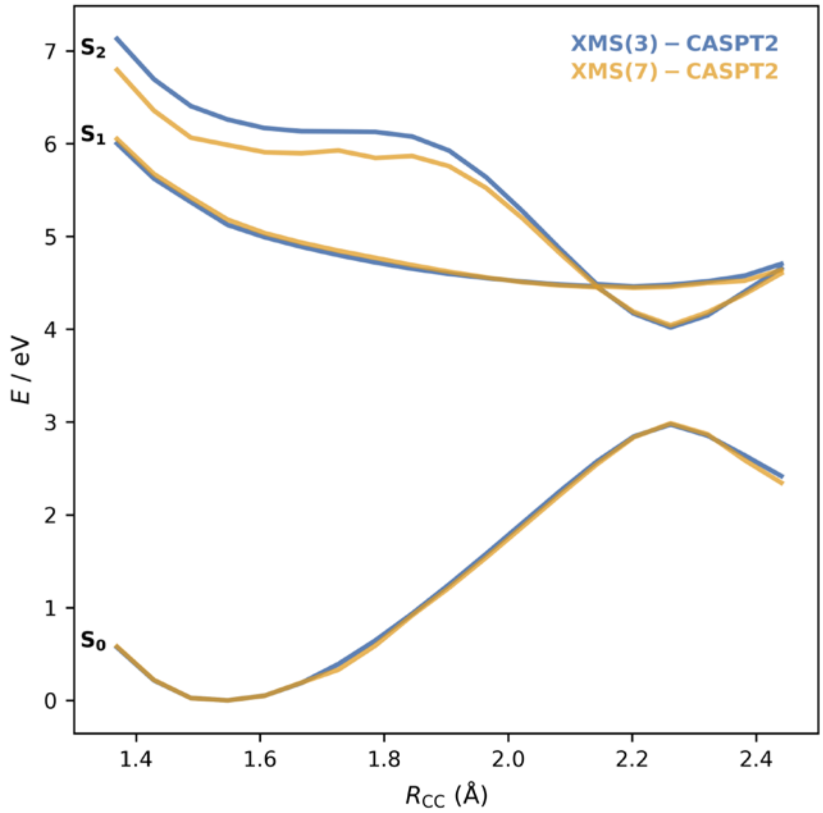

Figure S9. Comparison of the potential energy curves of the three lowest adiabatic states along the ring opening path of CHD in C_2 symmetry. The calculations were at the XMS(3)- CASPT2[6e,6o] (blue) and XMS(7)-CASPT2[6e,6o] (orange) levels of theory.

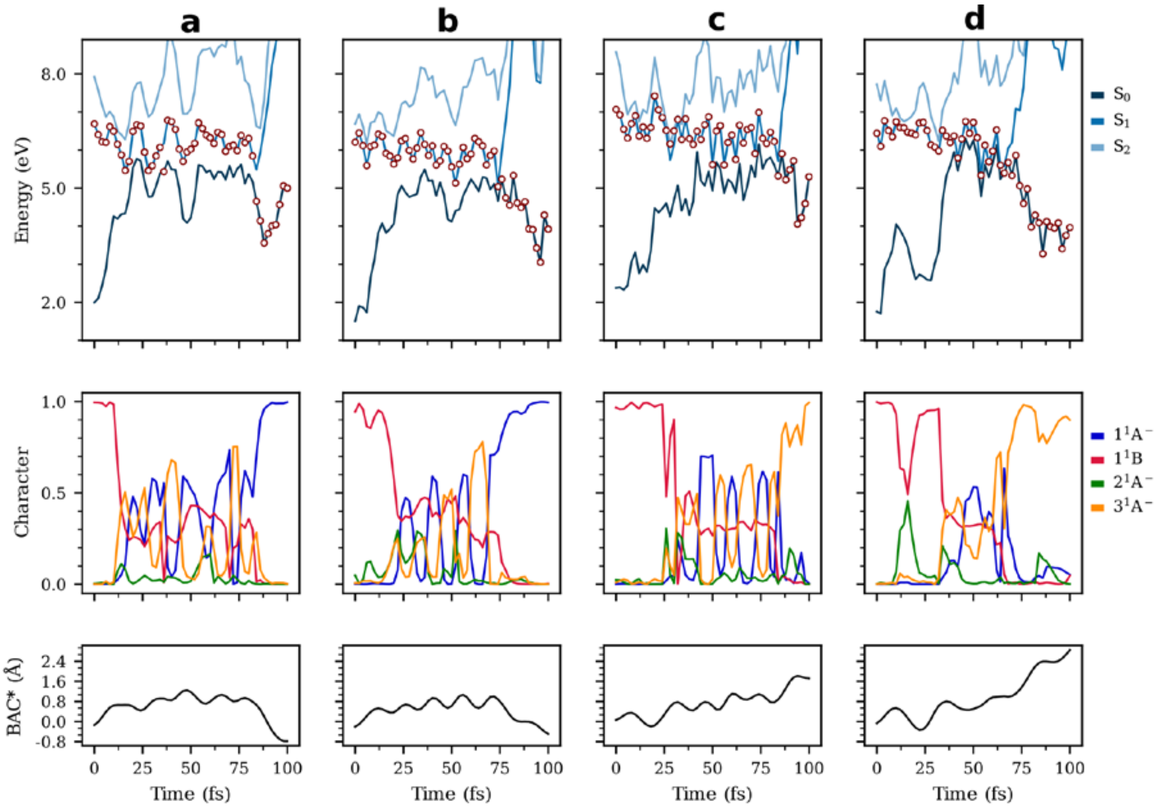

Figure S10. a,b Nonadiabatic dynamics trajectories leading to CHD. **c,d** Nonadiabatic dynamics trajectories leading to HT. **Top**, Time evolution of the potential energy of the electronic ground state S_0 (dark blue) and the two lowest excited states S_1 (blue) and S_2 (light blue). Dots mark the currently populated electronic state. **Middle**, Decomposition of the S_1 state in terms of four diabatic states, 1^1A^- (blue), 1^1B (red), 2^1A^- (green) and 3^1A^- (orange) along the trajectories. **Bottom**, Evolution of the extended bond alternation coordinate (BAC*) along the nonadiabatic trajectories.

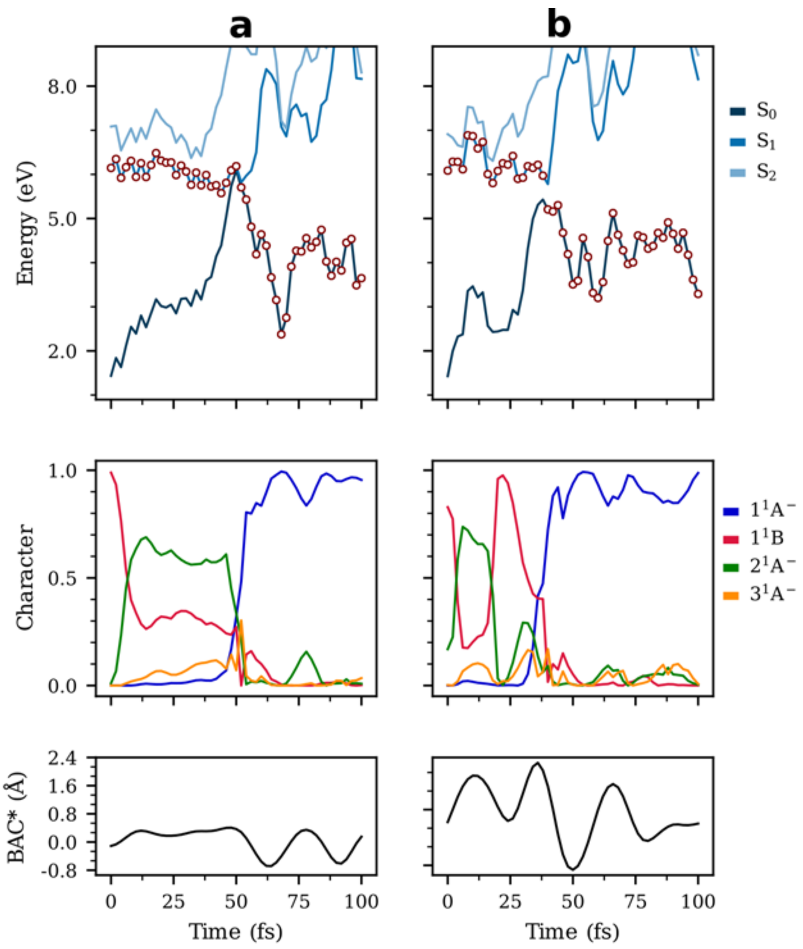

Figure S11. a,b, Nonadiabatic dynamics trajectories with short $R(C_1\text{-}C_6)$ bond distance and small BAC* at the time of deactivation to the ground state. **Top**, Time evolution of the potential energy of the electronic ground state S_0 (dark blue) and the two lowest excited states S_1 (blue) and S_2 (light blue). Dots mark the currently populated electronic state. **Middle**, Decomposition of the S_1 state in terms of four diabatic states, 1^1A^- (blue), 1^1B (red), 2^1A^- (green) and 3^1A^- (orange) along the trajectories. Notice the small contribution of the 3^1A^- state during the dynamics. The hop to the ground state occurs from the 2^1A^- state (a) and 1^1B (b) state.

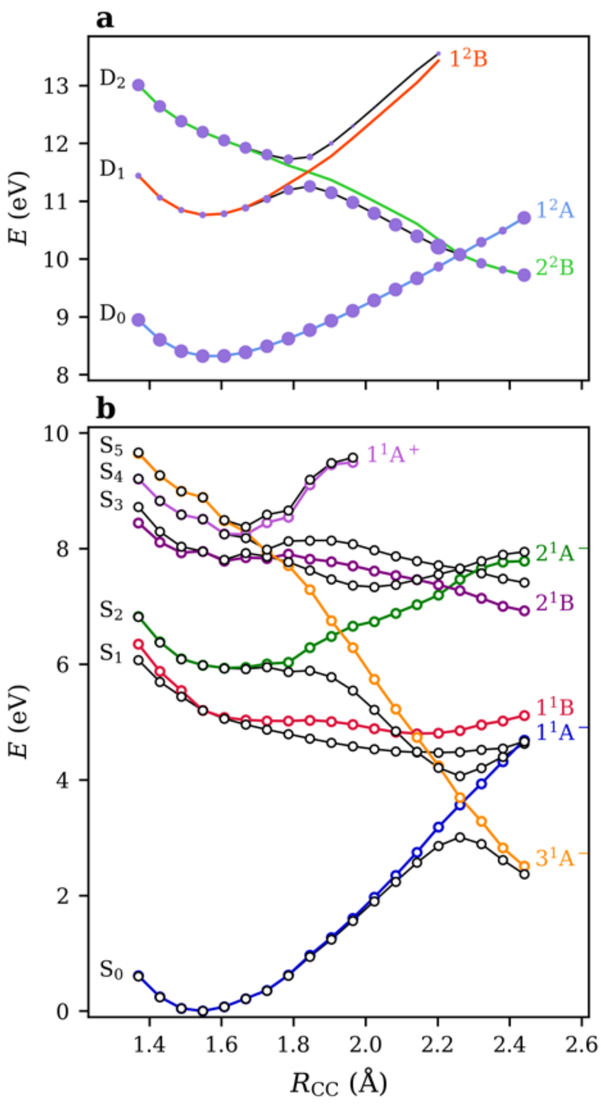

Figure S12. Adiabatic and diabatic potential energy surfaces of (**a**) CHD^+ and (**b**) CHD along the linearly interpolated ring-opening reaction coordinate in C_2 symmetry. The size of the circles on the cationic adiabatic surfaces is proportional to the square of the Dyson norm from the S_1 adiabatic state of CHD to the corresponding doublet state of the CHD^+ cation.

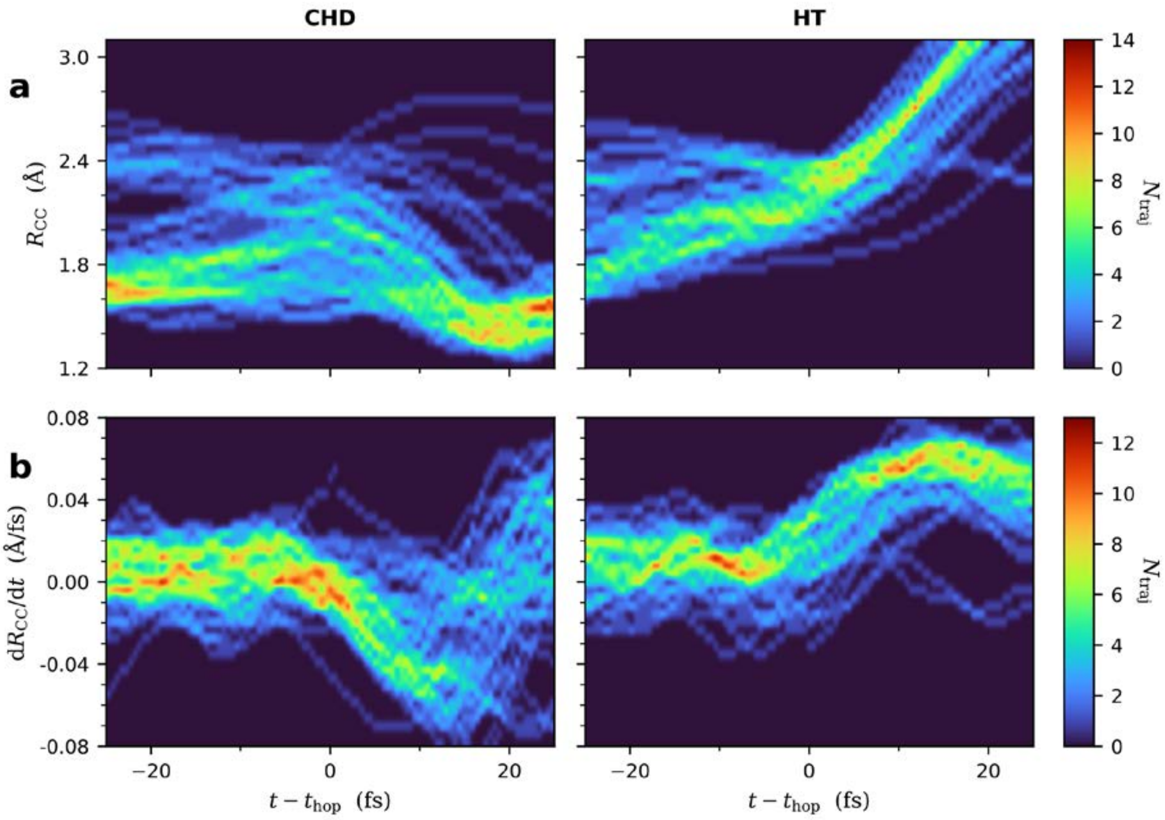

Figure S13. Evolution of the (**a**) R(C_1-C_6) bond length and (**b**) the relative velocity dR(C_1-C_6)/dt for the ensemble of nonadiabatic trajectories synchronised to reach the S_1/S_0 CoIn simultaneously at $t' = t - t_{hop} = 0$. Trajectories ending as (left) CHD and (right) HT are shown separately. The time derivative of the R(C_1-C_6) bond length is calculated as $\frac{\mathrm{d}R_{CC}}{\mathrm{d}t} = \frac{(\mathbf{R}_{C6}-\mathbf{R}_{C1})\cdot(\mathbf{v}_{C6}-\mathbf{v}_{C1})}{R_{CC}}$, where the vectors $\mathbf{R}_{Cn}$ and $\mathbf{v}_{Cn}$ contain the Cartesian coordinates and velocities of the Cn atoms.

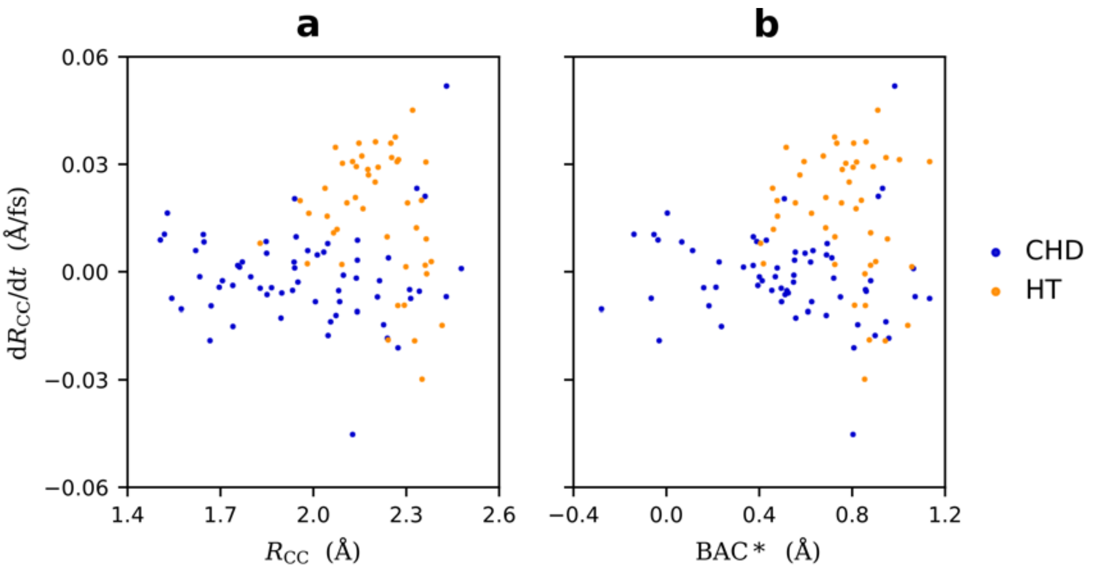

Figure S14. Distribution of S_1/S_0 CoIn geometries in (**a**) $R(C_1\text{-}C_6)$ - d$R(C_1\text{-}C_6)$/dt and (**b**) BAC*-d$R(C_1\text{-}C_6)$/dt spaces. Calculation of d$R(C_1\text{-}C_6)$/dt is described in the caption of Figure S13. Orange (blue) circles correspond to HT (CHD) trajectories.

5. References

[1] Svetina, C. *et al.* The low density matter (LDM) beamline at FERMI: optical layout and first commissioning. *J. Synchrotron Radiat.* **2015**, *22*, 538–543.
[2] Finetti, P *et al*. Optical setup for two-colour experiments at the low density matter beamline of FERMI. *J. Opt.* **2017**, *19***,** 114010.
[3] Zangrando, M. *et al.* The photon analysis, delivery, and reduction system at the FERMI@Elettra free electron laser user facility. *Rev. Sci. Instrum.* **2009**, *80***,** 113110.
[4] Zangrando, M. *et al.* Recent results of PADReS, the photon analysis delivery and reduction system, from the FERMI FEL commissioning and user operations. *J. Synchrotron Rad.* **2015**, *22***,** 565–570.
[5] Kruit, P. & Read, F.H. Magnetic field paralleliser for 2π electron-spectrometer and electron-image magnifier. *J. Phys. E: Sci. Instrum.* **1983**, *16***,** 313.
[6] Eland, J. H. D.; Feifel, R. Double ionisation of ICN and BrCN studied by a new photoelectron–photoion coincidence technique. *Chem. Phys.* **2006**, *327***,** 85-90.
[7] https://webbook.nist.gov/cgi/cbook.cgi?ID=C592574
[8] Kimura, K. *et al.*, *Handbook of HeI Photoelectron Spectra of Fundamental Organic Molecules*; Japan Scientific Societies Press, Tokyo, 1981; p. 68.
[9] Shiozaki, T.; Gyorffy, W.; Celani, P.; Werner, H-J. Extended multi-state complete active space second-order perturbation theory: energy and nuclear gradients. *J. Chem. Phys.* **2011**, *135***,** 081106.
[10] Vlaisavljevich, B.; Shiozaki, T. Nuclear energy gradients for internally contracted complete active space second-order perturbation theory: multistate extensions. *J. Chem. Theory Comput.* **2011**, *12***,** 3781–3787.
[11] Park, J. W.; Shiozaki, T. Analytical derivative coupling for multistate CASPT2 theory. *J. Chem. Theory Comput.* **2017**, *13***,** 2561–2570.
[12] Shiozaki, T. BAGEL: Brilliantly advanced general electronicstructure library. *Wiley Interdiscip. Rev.: Comput. Mol. Sci.* **2018**, *8***,** el1311.
[13] Shiozaki, T. BAGEL: Brilliantly Advanced General Electronicstructure Library. http://www.nubakery.org under the GNU General Public License.
[14] Yan, Y. J.; Fried, L. E.; Mukamel, S. Ultrafast Pump-Probe Spectroscopy: Femtosecond Dynamics in Liouville Space. *J. Phys. Chem.* **1989**, *93***,** 8149–8162.
[15] Yan, Y. J.; Mukamel, S. Femtosecond pump-probe spectroscopy of polyatomic molecules in condensed phases. *Phys. Rev. A* **1990**, *14***,** 6485–6504.
[16] Gelin, M. F. *et al.* Ab initio surface-hopping simulation of femtosecond transient absorption pump-probe signals of nonadiabatic excited state dynamics using the doorway-window representation. *J. Chem. Theory Comput.* **2021**, *17***,** 2394–2408.
[17] Piteša, T. *et al.* A combined surface-hopping, Dyson orbital and B-spline approach for the computation of time-resolved photoelectron spectroscopy signals: the internal conversion in pyrazine. *J. Chem. Theory Comput.* **2021**, *17***,** 5098–5109.
[18] Tully, J. C. Molecular dynamics with electronic transitions. *J. Chem. Phys.* **1990**, *93***,** 1061–1071.
[19] Sapunar, M.; Piteša, T.; Davidović, D.; Došlić, N. Highly efficient algorithms for CIS type excited state wave function overlaps. *J. Chem. Theory Comput.* **2019**, *15***,** 3461–3469.
[20] Piteša T.; Alešković, M.; Becker, K.; Basarić, N.; Došlić, N. Photoelimination of nitrogen from diazoalkanes: involvement of higher excited singlet states in the carbene formation. *J. Am. Chem. Soc.* **2020**, *142***,** 9718–9724.
[21] Granucci, G.; Persico, M.; Toniolo, A. Direct semiclassical simulation of photochemical processes with semiempirical wave functions. *J. Chem. Phys.* **2001**, *114***,** 10608–10615.
[22] Granucci, G.; Persico, M. Critical appraisal of the fewest switches algorithm for surface hopping. *J. Chem. Phys.* **2007**, *126***,** 134114.
[23] Bonačić-Koutecký, V.; Mitrić, R. Theoretical Exploration of Ultrafast Dynamics in Atomic Clusters: Analysisand Control. *Chem. Rev.* **2005**, *105***,** 11–65.
[24] Werner, H.-J.; Knowles, P. J.; Knizia, G.; Manby, F. R.; Schütz, M. Molpro: a general-purpose quantum chemistry program package. *WIREs Comput. Mol. Sci.* **2012**, *2*, 242–253.

[25] Shu, Y.; Varga, Z.; Kanchanakungwankul, S.; Zhang, L.; Thrular, D. G. Diabatic States of Molecules. *J. Phys. Chem. A* **2022**, *126*, 992–1018.
[26] Polyak, I.; Hutton, L.; Crespo-Otero, R.; Barbatti, M.; Knowles, P. J. Ultrafast photoinduced dynamics of 1,3-cyclohexadiene using XMS-CASPT2 surface hopping. J. Chem. Theory Comput. **2019**, *15*, 3929–3940.
[27] Simah, D.; Hartke, B.; Werner, H.-J. Photodissociation dynamics of H_2S on new coupled ab initio potential energy surfaces. *J. Chem. Phys.* **1999**, *111*, 4523-4534.
[28] Finley, J.; Malmqvist, P.; Roos, B. O.; Serrano-Andrés, L. The multi-state CASPT2 method. *Chem. Phys. Lett.* **1998**, *288*, 299−306.
[29] Landry, B. R.; Falk, M. J.; Subotnik, J. E. The correct interpretation of surface hopping trajectories: How to calculate electronic properties. *J. Chem. Phys.* **2013**, *139*, 211101.
